# Urinary Proteome Profiling by Several Methods Identifies Titin as the Most-Differentiating Noninvasive Urinary Biomarker of Disease Severity in Becker Muscular Dystrophy

**DOI:** 10.3390/proteomes14030043

**Published:** 2026-08-25

**Authors:** Kimchi K. Le, Emily H. Canessa, Corrine M. Stahura, Jenna Mayers, Katie A. Edwards, Marissa Barbieri, Eric P. Hoffman, Yetrib Hathout

**Affiliations:** Department of Pharmaceutical Sciences, School of Pharmacy and Pharmaceutical Sciences, Binghamton University—SUNY, Binghamton, NY 13790, USA; kle5@binghamton.edu (K.K.L.); ecanessa@binghamton.edu (E.H.C.); cstahur1@binghamton.edu (C.M.S.); jmayers2@binghamton.edu (J.M.); kedwards@binghamton.edu (K.A.E.); barbieri@binghamton.edu (M.B.); ehoffman@binghamton.edu (E.P.H.)

**Keywords:** Becker muscular dystrophy, biomarkers, N-terminal titin, urine samples, proteomics, non-invasive

## Abstract

Background: Beckers muscular dystrophy (BMD) is a clinically heterogeneous dystrophinopathy caused by in-frame mutations in the dystrophin gene, resulting in variable disease severity. This variability limits the effectiveness of standardized functional measures for tracking progression. Circulating biomarkers, including urinary titin fragments generated during muscle injury, offer a promising non-invasive approach for assessing disease status. Methods: Mass spectrometry-based urinary proteomic profiling identified titin fragments as candidate biomarkers of muscle injury in BMD. These titin fragments were validated by two independent methods, targeted mass spectrometry and ELISA, using subsets of ambulatory BMD, non-ambulatory BMD and age matched healthy volunteers. Results: Urinary titin levels were significantly elevated by 4.56-fold and 2.32-fold in ambulatory and non-ambulatory BMD patients, respectively, compared with healthy controls. Titin levels also distinguished ambulatory from non-ambulatory BMD patients, demonstrating potential utility for monitoring disease progression and therapeutic response. Conclusions: Urinary titin fragments represent a promising non-invasive biomarker for BMD, with potential applicability to related neuromuscular disorders and clinical trials evaluating disease progression and treatment efficacy.

## 1. Introduction

Becker muscular dystrophy (BMD) is an X-linked recessive inherited disease typically caused by in-frame mutations in the dystrophin gene that lead to the expression of partially functional dystrophin [1,2]. Despite its clinical significance, BMD has historically received less attention and translational research focus compared to Duchenne muscular dystrophy (DMD). This disparity is largely due to the longstanding perception of BMD as a “mild” form of the muscular dystrophies [3,4]. However, the BMD population is very heterogeneous with symptoms ranging from relatively mild muscle weakness allowing individuals to walk into their 50s to severe early onset weakness resulting in loss of ambulation typically before the ages of 12–15 [1]. Because of this heterogeneity, there is a lack of standardized and validated outcome measures for BMD as compared with DMD [5]. Many clinical assessment tools and trial endpoints were originally developed for DMD and may not fully capture the slower progression or distinct clinical features of BMD. As a result, subtle but clinically meaningful changes in function may go undetected in research settings.

Circulating biomarkers, on the other hand, are increasingly shedding light on assessing the severity and progression of BMD [6]. If validated, they could become useful tools for assessing disease severity, monitoring disease progression, evaluating response to therapies, and enhancing overall treatment strategies. Further biomarker studies are therefore needed to support more personalized treatment approaches and ultimately improve outcomes and quality of life for individuals with BMD.

BMD patients generally share similar circulating biomarkers as DMD patients but typically at different levels. Both disorders are characterized by elevated serum creatine kinase (CK), an enzyme that leaks out due to muscle damage, and is often used as a pre-diagnostic biomarker for DMD and BMD [7]. However, it has limited utility in assessing disease severity and progression in these dystrophinopathies because it does not correlate with clinical outcome measures [6,8].

Other biomarkers measured in serum samples such as creatine-to-creatinine ratios and titin have been recently shown to correlate with disease severity in BMD [6,9]. Furthermore, the N-terminal fragment of titin detected in urine has been shown to be a key indicator of muscle injury in a number of muscular dystrophies and non-muscle disease including DMD [10,11], adult spinal muscular atrophy (SMA) [12], myotonic dystrophy (DM1) [13], Fukuyama congenital muscular dystrophy (FCMD) [14], sarcopenia in diabetes [15], acute cerebral stroke [16], amyotrophic lateral sclerosis (ALS) [17], and patients with intensive care-acquired weakness [18]. Its clinical utility has been demonstrated based on its ability to assess muscle damage across different muscles and its response to dystrophin restoring therapies such as AAV-gene therapy [19].

In this study, we performed urinary proteome profiling to identify potential non-invasive protein biomarkers for assessing muscle injury in individuals with BMD, across a broad range of disease severities and ages. Titin fragments generated through titin proteolytic cleavage during muscle degeneration were consistently detected in higher levels in the urine of individuals with BMD than in healthy controls. Specifically, urinary levels of the N-terminal titin fragment were 4.56-fold and 2.32-fold higher in ambulatory and non-ambulatory individuals with BMD, respectively, compared with healthy controls.

## 2. Materials and Methods

### 2.1. Study Participants, Demographics, and Urine Collection

This biomarker study was approved by the Institutional Review Board at Binghamton University. Information about the study was disseminated through Parent Project Muscular Dystrophy (PPMD), BMD-specific support groups and forums on Facebook, physicians who treat patients with BMD, and through the Muscular Dystrophy Associations email listserv and outreach publications. Interested participants or parents or guardians of the participant contacted the clinical coordinator to express interest in the study. Participants ranged in age from 6 to 73 years. After providing informed consent (or parental consent and assent, when applicable), participants were asked to provide a copy of their DNA diagnostic bloodwork to confirm a diagnosis of BMD. A mobile phlebotomist collected blood and urine samples at the participants’ homes. Urine samples were collected from 50 participants, stratified at the time of collection as ambulatory BMD (*n* = 33) and non-ambulatory BMD (*n* = 17). Immediately after collection, urine samples were filtered by the phlebotomist through a 0.22 μm Millipore Nylon filter (Sigma-Aldrich, Inc., St. Louis, MO, USA) into 15 mL Falcon^TM^ High Clarity Polypropylene Conical tubes (Thermo Fisher Scientific, Chicago, IL, USA), placed into biohazard bags, and shipped on dry ice via FedEx to Binghamton University School of Pharmacy and Pharmaceutical Sciences. To minimize repeated freeze–thaw cycles, the samples were aliquoted into 1 mL working volumes upon arrival and stored at −80 °C until subsequent processing and analysis. All samples were processed and analyzed using the same procedures to reduce variability associated with sample handling.

Healthy control urine samples were collected from local volunteers who were recruited through an advertisement in the university e-communications daily newsletter. Upon completion of informed consent, the participants visited the School of Pharmacy building, providing up to 100 mL of urine. The healthy control volunteers (age range from 24 to 64) confirmed that they had not taken any glucocorticoids or other medications within the past 90 days. Urine samples were collected from 11 healthy volunteers and processed as described above.

The following patient-reported outcomes were collected for BMD patient samples: age, age of onset of symptoms, age of loss of ambulation, exon mutation type, and medication regimen. For healthy controls, age and medication regimen were collected.

### 2.2. Study Design: Different Methods and Sample Numbers Used for Measurement of Urinary Proteins

This study was structured into two phases: discovery and validation. We used two independent quantitative proteomics methods for the discovery of candidate BMD biomarkers in the urine samples: the Label-Free Quantification (LFQ) and the tandem mass tag (TMT)-based quantification. The LFQ methods were performed for initial preliminary assessment of the overall urine proteome by focusing on a small cohort of samples (ambulatory BMD (*n* = 5), non-ambulatory BMD (*n* = 4), healthy controls (*n* = 5)). This was followed by TMT-based quantification also performed in a small cohort (ambulatory BMD (*n* = 3), non-ambulatory BMD samples (*n* = 3), and age-matched healthy controls (*n* = 3)), to confirm our initial findings in an isolated method. In both methods, urinary N-terminal titin appeared to be a candidate due to its significant difference not only between disease severity and control (aBMD/Cnt or naBMD/Cnt) but also between the disease severities (aBMD/naBMD).

This prompted further validation of urinary N-terminal titin using targeted mass spectrometry (ambulatory BMD (*n* = 21), non-ambulatory BMD (*n* = 6), age-matched healthy controls (*n* = 3)), a commercially available ELISA (ambulatory BMD (*n* = 20), non-ambulatory BMD (*n* = 10), age-matched healthy controls (*n* = 10)), and Western blot analysis (ambulatory BMD (*n* = 3), non-ambulatory BMD (*n* = 3), age-matched healthy controls (*n* = 3)). The differing numbers of samples in each cohort were attributed to limitations in sample availability and quality for analysis.

In our methods of analysis of our quantitative results using the commercially available ELISA, urinary N-terminal titin levels were normalized using total protein. Normalization in the context of this manuscript is a ratio-based parameter used to allow for the co-temporal background variation in actual level of all urinary protein biomarkers due to normal fluctuations of urine concentration/dilution.

#### 2.2.1. Label-Free Quantitative Proteomics

Urine aliquots containing 15 μg of total protein were vacuum-centrifuged to approximately 100 μL in Eppendorf LoBind tubes (Fisher Scientific, Waltham, MA, USA) to prevent protein loss. Subsequently, 25 μL of 0.5% SDS was added, and samples were heated at 40 °C for 15 min with shaking at 500 rpm. Protein disulfide bonds were reduced by adding 7 μL of 200 mM dithiothreitol (DTT) (Pierce Biotechnology, Woburn, MA, USA) prepared in 25 mM ammonium bicarbonate (Thermo Fisher Scientific, Waltham, MA, USA), followed by incubation at 60 °C for 1 h with shaking at 500 rpm. Reduced cysteines were alkylated by adding 44 μL of 200 mM iodoacetamide (IAA) Sigma Aldrich, Burlington, MA, USA) in 25 mM ammonium bicarbonate and incubating for 30 min at 30 °C while shaking in the dark. Proteins were precipitated by adding 900 μL (approximately 4–5 volumes) of ice-cold 100% acetone (−20 °C) (Sigma Aldrich, Burlington, MA, USA), followed by centrifugation at 17,000× *g* for 30 min at 4 °C. Samples were then stored at −80 °C for 20 min to enhance protein precipitation and centrifuged again at 17,000× *g* at 4 °C for 10 min. The acetone was carefully removed by decanting or pipetting without disturbing the pellet. Pellets were air-dried for 2 min at 37 °C. Proteins were digested by adding 100 μL of 25 mM ammonium bicarbonate and 3 μL of 0.10 μg/μL sequencing grade trypsin solution (Promega, Madison, WI, USA), followed by overnight incubation at 37 °C with shaking at 500 rpm. Digestion was stopped by adding 5 μL of 2.5% trifluoroacetic acid (TFA) (Thermo Fisher Scientific, Waltham, MA, USA) and 200 μL of 0.1% TFA prior to fractionation.

Each sample was fractionated using Pierce™ High-pH Reversed-Phase Peptide Fractionation columns and solutions provided in the kit (Thermo Fisher Scientific, Waltham, MA, USA). Peptides were sequentially eluted with solutions containing 7.5%, 12.5%, 17.5%, and 50% acetonitrile (ACN) in 0.1% triethylamine. The resulting eluates were vacuum-dried at 45 °C until completely dry and stored at −80 °C until mass spectrometry analysis.

#### 2.2.2. Tandem Mass Tag (TMT)

All the reagents used for TMT preparation were from the TMT Sixplex Isobaric Mass Tagging Kit (Thermo Fisher Scientific, Waltham, MA, USA). Aliquots of 100−350 μL containing 15 μg of total proteins from each sample were vacuum-dried to 30 μL and denatured with 20 μL of 1% sodium dodecyl sulfate (SDS) (J. T. Baker, Radnor, PA, USA) and heated at 40 °C for 15 min at 450 rpm. Disulfide bonds were reduced by adding 5 μL of 200 mM Tris(2-carboxyethyl) phosphine (TCEP) (Sigma Aldrich, Burlington, MA, USA) followed by incubation at 55 °C for 1 h at 450 rpm. Free thiols were then alkylated by adding 5 μL of 375 mM iodoacetamide (IAA) followed by incubation at 40 °C for 1.5 h in the dark at 450 rpm. Proteins were then precipitated by adding 360 μL of ice-cold 100% acetone (−20 °C), followed by centrifugation at 15,000 rpm for 30 min at 4 °C. For acetone washing, the supernatant was removed slowly, leaving 10–20 μL in the tube to not disturb the pellet, 20–30 μL of HPLC H_2_O (Thermo Fisher Scientific, Waltham, MA, USA) was added to the sample to complete a total volume of 60 μL. Samples were then stored at −20 °C for 20 min, followed by adding 360 μL of ice-cold 100% acetone (−20 °C) before repeating the centrifugation step at 15,000 rpm for 30 min at 4 °C. The precipitate from each sample was kept at −20 °C overnight (~16 h). The mixture was then centrifuged again the next day at 15,000 rpm for 20 min at 4 °C and the sample went through the same acetone wash for an additional two times to get rid of the remaining salt completely; after the last centrifugation step, the supernatant was removed leaving behind 10–20 μL to not disturb the pellet. The remaining supernatant was then dried on a heat block with the cap open for approximately 15 min at 45 °C. Then, 100 μL of 50 mM triethyl ammonium bicarbonate (TEAB) (Sigma Aldrich, Burlington, MA, USA) was added to the protein pellet and vortexed for 1 min. For protein digestion, MS Grade Trypsin Protease (Thermo Fisher Scientific, Waltham, MA, USA), corresponding to 1 μg of trypsin per 100 μg of total protein, was added to each sample. The samples were incubated for 4 h at 37 °C with sharking at 450 rpm, with gentle vortexing and brief centrifugation using a tabletop centrifuge every hour. An additional aliquot of trypsin was then added to achieve a final enzyme-to-protein ratio of 1:50 (*w*/*w*), followed by overnight digestion at 37 °C with shaking at 450 rpm. For checkpoint purposes, 10 μL of sample were dried down in Lo-Bind tubes, reconstituted in 0.1% formic acid, and loaded onto the mass spectrometer to test for digestion efficiency. Another 10 μL of sample, stored in Lo-Bind tubes, was processed through a Quantitative Fluorometric Peptide Assay (Thermo Scientific, Waltham, MA, USA) at a ¼ dilution (adding 30 μL HPLC H_2_O), to confirm equal amounts of peptide material in each sample. The remainder of the sample was frozen at −80 °C in Lo-Bind tubes for TMT mass tagging following the manufacturer’s protocol (Thermo Fisher Scientific, Waltham, MA, USA).

After TMT labeling samples with different mass tags, samples were combined into one single tube, dried by vacuum centrifugation, resuspended in 300 μL of 0.1% TFA and subjected to high pH reversed phase fractionation. In total, 10 consecutive fractions were collected with elution buffers gradually increasing with ACN between 5%, 7.5%, 10%, 12.5%, 15%, 17.5%, 20%, 50%, 65%, and 80%, all in triethylamine. The flow through (FT), wash (W), and 10 collected fractions were dried using a SpeedVac vacuum concentrator (Thermo Fisher Scientific, Waltham, MA, USA) the same day or stored overnight to load on the vacuum centrifuge the following morning and stored at −20 °C until nano LC−MS/MS analysis.

#### 2.2.3. Liquid Chromatography-Based Mass Spectrometry (LC-MS/MS) and Data Processing

Each dried peptide sample was resuspended in 10–20 μL of 0.1% formic acid (Pierce Biotechnology, Woburn, MA, USA) and transferred to an autosampler vial for mass spectrometry analysis. Samples (2 μg aliquots) were loaded onto a nano-LC system, Dionex UltiMate 3000 RS UPLC (Thermo Fisher Scientific, Chicago, IL, USA) coupled to a Q Exactive HF-X Hybrid Quadrupole-Orbitrap Mass Spectrometer (Thermo Fisher Scientific, Chicago, IL, USA) and processed in order of high pH fractionation elutions, four for LFQ per sample and 12 for TMT per condition comparison. A two-hour gradient was used with mobile phase A (0.1% formic acid in water) and mobile phase B (0.1% formic acid in 80% acetonitrile). An EASY-Spray LC Column (75 μm inner diameter, 50 cm length, 100 Å pore size, 2 μm particle size (Thermo Scientific, Waltham, MA, USA)) was equilibrated with 98% mobile phase A and 2% mobile phase B for 8 min, followed by a 5–30% B gradient over 87 min, 30–100% B over 5 min, 100% B for 5 min, 100–2% B over 5 min, and for re-equilibration at 2% B for 10 min. Data-dependent acquisition (DDA) was performed at a resolution of 120,000 over a scan range of 375–1600 *m*/*z*. Each cycle consisted of one full MS scan followed by 20 MS/MS scans. MS/MS spectra were acquired at 27 eV normalized collision energy, with a spray voltage of 4.0 kV and a column temperature of 35 °C.

Protein identification was performed against the human proteome database (UniProtKB/Swiss-Prot; TaxID 9606, release 7 May 2017; 25,496 entries) using the Sequest algorithm implemented in Proteome Discoverer (Thermo Fisher Scientific, Waltham, MA, USA). A precursor mass tolerance of ±10 ppm and a fragment mass tolerance of ±0.05 Da were used. The following were set as variable modifications: carbamidomethylation of cysteine, oxidation of methionine, acetylation of peptide N-termini. Peptide-spectrum matches (PSMs) and peptides were filtered using a target–decoy strategy at a strict 1% false discovery rate (FDR). Label-free quantitative proteomics were performed using PSMs as previously described [20].

#### 2.2.4. TMT Quantitative Proteomics: Database Searching and Data Processing

TMT-based quantitative proteomics was performed in the same manner as for label-free proteomics, with the exception that TMT 6-plex modifications on lysine side chains and peptide N-termini were included as static modifications. Peptide-spectrum matches were filtered using a target–decoy strategy to maintain a false discovery rate (FDR) below 1% at both the peptide and protein levels. Reporter ion–based quantification was performed in Proteome Discoverer using the TMT 6-plex workflow. Reporter ions (*m*/*z* 126, 127, 128, 129, 130, and 131) were quantified with a mass tolerance of 0.05 Da, and the most confident centroid peak was used for integration. Only unique peptides were used for quantification, and spectra containing any missing reporter ion channels were excluded. Isotopic impurity corrections provided by the manufacturer were applied to the reporter ion intensities. A minimum signal-to-noise ratio of 1.5 was required for each channel. Normalization was performed using the “total peptide amount” normalization mode in the consensus workflow. The overall peptide abundance in each sample was calculated by summing the reporter ion intensities of the corresponding protein across all fractions.

#### 2.2.5. Validation of Titin as a Biomarker Candidate Using Targeted Mass Spectrometry

Stable isotope-labeled proteins including titin protein were generated through culture of immortalized human myoblast cells [21] in stable isotope-labeled culture media (SILAC) where Arg and Lys were replaced by ^13^C_6_–Arg and ^13^C_6_,^15^N_2_–Lys (Thermo Fisher Scientific, Waltham, MA); the methods used to generate these human myotubes have been previously described in Canessa et al., 2020 [22]. Total protein extracts containing stable isotope titin were used as internal standard to relatively quantify levels of titin fragments in urine samples from BMD (ambulatory *n* = 21, non-ambulatory *n* = 6) and age-matched healthy controls (*n* = 3). Each urine sample containing 15 µg total protein was spiked with 2.0 μg SILAC-labeled myotube extract. The following titin tryptic peptide VPAPVEIPVTPPTLVSGLK (Light *m*/*z* = 957.5692++, Heavy *m*/*z* = 961.5763++; RT:105.19) was targeted for quantification using a PRM method.

Due to sample complexity, absolute quantification of titin in SILAC myotubes was performed using a 2 h data-dependent acquisition (DDA) method for peptide discovery and a 2 h parallel reaction monitoring (PRM) method for targeted quantification, with samples processed in duplicate. Both LC-MS/MS methods used identical chromatographic conditions. Mobile phase A consisted of 0.1% formic acid in water and mobile phase B of 0.1% formic acid in 80% acetonitrile. Peptides (2 μg) were separated on an EASY-Spray™ LC column 75 μm × 500 mm, 2 μm particles, 100 Å pore size; Thermo Scientific, Waltham, MA, USA) at 0.300 μL/min. The gradient was: 2% B for 8 min; 5–30% B over 87 min; 30–100% B for 5 min; 100% B for 5 min; 100–2% B for 5 min; and 2% B for 5 min. DDA full MS scans were acquired at 120,000 resolution (375–1600 *m*/*z*), followed by 20 MS/MS scans at 30,000 resolution and 27 eV collision energy. PRM MS2 scans were acquired at 60,000 resolution, with all other parameters unchanged (4.0 kVV ion spray, 35 °C column temperature). Retention times and precursor *m*/*z* values from DDA runs were used to generate PRM methods for selected peptides. Protein identification was performed against the human SwissProt database (TaxID 9606; v2017-07-05) using the Sequest algorithm in Proteome Discoverer 2.2 (Thermo Fisher Scientific, Waltham, MA, USA) and resulting .raw files were imported into Skyline for quantification.

#### 2.2.6. Validation of Titin as a Biomarker Candidate Using Commercially Available ELISA Assay

A urinary ELISA assay, targeting the N-terminal fragment of titin protein, was purchased from Immuno-Biological Laboratories, INC (IBL, Minneapolis, MN, USA) and used to confirm the mass spectrometry data. This ELISA assay was previously used in numerous articles to measure levels of titin in urine samples [11,19,23]. The standard curve provided by the manufacturer set a range of 46.88~3000 pmol/L, was converted to ng/mL for analysis using the first 200 amino acids of the titin protein as the molecular weight for calculations. Aliquots of 10 µL of urine samples from BMD subjects (*n* = 30) and age-matched healthy controls (*n* = 10) were diluted at 1:10; samples and standards were plated in duplicate at 100 µL/well and processed for the ELISA assay as described in the manufacturer’s protocol. Samples were measured through a standard plate reader measuring absorbance at 450 nm. Analysis was done based on the manufacturer’s instructions using quadratic regression of double-logarithm conversion.

Data were normalized to total protein concentration in urine, as creatinine levels are known to fluctuate in individuals with disease severity in DMD and BMD [24]. For total protein quantification, urine samples were prepared using a Compat-Able Assay (Thermo Fisher Scientific, Waltham, MA, USA) with 0.5% SDS resolubilization prior to BCA assay analysis. This preparation step removed particulates that could interfere with the BCA assay, as well as salts, reducing agents, detergents, and intrinsic color from the urine samples. Total protein concentrations were then determined using MicroBCA/BCA assays (Thermo Fisher Scientific, Waltham, MA, USA). Other methods for normalization (creatinine, specific gravity) are provided in the Supplemental Material.

#### 2.2.7. Western Blot Analysis

To verify presence of N-terminal titin in the BMD urine samples, Western blotting was performed on urine samples from healthy controls (*n* = 3), ambulatory BMD (*n* = 3), and non-ambulatory BMD (*n* = 3) patients. Samples were concentrated down to 15 µg at 100 µL, desalted using Zeba Spin Desalting Columns 7K MWCO (Thermo Scientific, Waltham, MA, USA), centrifuged, acetone-precipitated, resolubilized in 0.5% SDS, and separated on NuPAGE^TM^ 4–12% Bis-Tris gels (Thermo Fisher Scientific, Waltham, MA, USA). Proteins were transferred overnight to nitrocellulose membranes, blocked, and incubated with anti-Titin 6H-5 antibody (Abnova Corporation, Taipei, Taiwan) overnight at 4 °C. After washes, membranes were incubated with ECL Anti-mouse IgG horseradish peroxidase linked whole antibody (originally GE Healthcare UK Limited, now Cytiva Life Sciences, Marlborough, MA, USA), and signals were detected using ECL Prime Western Blotting Detection Reagents (Cytiva Life Sciences, Marlborough, MA, USA) on an Azure Biosystems Imager (Azure Biosystems, Dublin, CA, USA).

#### 2.2.8. Statistical Analysis

The raw mass spectrometry proteomics data supporting reported results in this study have been deposited to the ProteomeXchange Consortium via the PRIDE52 partner repository. GraphPad version 11.0.0 was used for analyzing the data. Volcano plots were made using R statistical software version 4.4.3, the tidyverse package including ggplot2 for visualization and data manipulation to compare the significant proteins found among the disease severities for the label-free proteome discovery phase. Significant biomarkers were categorized based on a fold change (FC) being a non-negative ratio/fraction unless otherwise stated with a cut-off ≥1.5, ≤−1.5; *p*-value ≤ 0.05. For visualization, non-negative abundance ratios were converted to a signed fold-change metric. Ratios ≥1 were retained as positive values, whereas ratios <1 were transformed as −1/ratio. Statistical methods included multi-linear analysis, two-tailed *t*-tests, correlation analysis, one-way ANOVA, and post hoc analysis when necessary (Tukey’s, Dunnett’s T3, and Holm–Sidak’s). Targeted PRM analysis was log2-transformed to adjust for skewness of the data.

## 3. Results

### 3.1. Quantitative Proteome Profiling of BMD and Healthy Control Urine Samples

#### 3.1.1. Label-Free—Quantitative (LFQ) Proteomics Data

A total of 1748 proteins were identified and analyzed after processing 14 urine samples, which included healthy control volunteers (*n* = 5), ambulatory BMD (*n* = 5), and non-ambulatory BMD (*n* = 4). After removing proteins with missing data across all samples and excluding keratin contaminants, 795 proteins were retained for further analysis. Of these, 106 were significantly elevated (≥1.5-fold, *p*-value ≤ 0.05) and none were significantly decreased in urine samples of ambulatory BMD compared to healthy controls (Supplemental Figure S1a, Supplemental Table S1) while 25 proteins were found significantly increased (≥1.5-fold, *p*-value ≤ 0.05) and 2 were significantly decreased (≤−1.5-fold, *p*-value ≤ 0.05) in non-ambulatory BMD compared to healthy controls (Figure S1b, Supplemental Table S2). Because urine contains proteins shed from different organs, we focused on muscle-enriched proteins. Tissue-specificity mapping, according to the UniProt and the Human Proteome Atlas, of all significantly elevated proteins that were found in both ambulatory and non-ambulatory BMD patients, identified 6 potentially muscle-enriched proteins. These are listed in Table 1, along with their fold changes and *p*-values in both BMD groups relative to controls.

**Figure 1:**
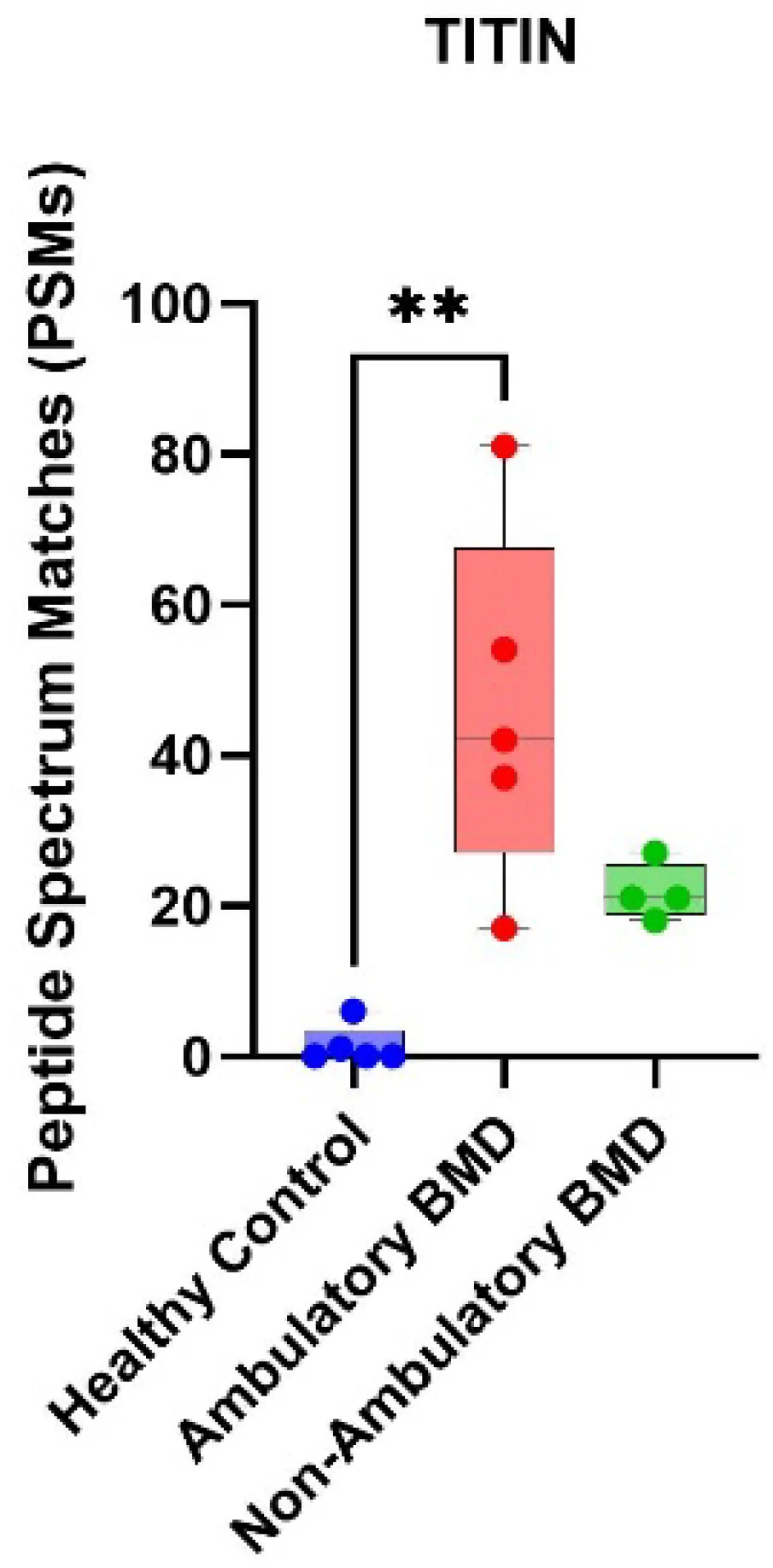
Peptide-spectrum matches (PSMs) of titin in BMD urine samples: box plot showing levels of urinary titin measured using label-free proteomic method. Healthy controls (*n* = 5); ambulatory BMD (*n* = 5), non-ambulatory BMD (*n* = 4) ** indicates significant adjusted *p*-value of 0.0013; reported using Tukey’s HSD post hoc analysis.

As shown in Figure 1, a one-way ANOVA revealed a significant difference in the levels of urinary titin between groups (*F* = 12.04, *R*^2^ = 0.6865, *p*-value = 0.0017). Tukey’s HSD post hoc test indicated significantly higher urinary titin levels in aBMD versus Cnt: (mean difference = −44.80, (95% CI −69.48 to 20.1) adj. *p*-value = 0.0013). A secondary post hoc analysis using healthy controls as the reference group was conducted with Dunnett’s T3, indicating significantly higher levels of aBMD versus Cnt and naBMD versus Cnt (see Supplemental Figure S2). Additionally, a significant FC and *p*-value were determined between aBMD and healthy controls using *t*-test, FC: 33, *p*-value = 0.0029; a significant FC: 15.54 and *p*-value = 2.81 × 10^−5^ was also determined between non-ambulatory BMD versus healthy controls. This significant change in urinary titin with disease stage makes it a good biomarker candidate for validation using orthogonal methods as described below.

#### 3.1.2. TMT—Quantitative Proteomics Data

In addition to our discovery phase using LFQ proteomics, a 6-plex TMT methodology was used to measure levels of proteins in urine samples from ambulatory BMD samples (*n* = 3), non-ambulatory BMD samples (*n* = 3), and age-matched healthy controls (*n* = 3). To maintain cohesion in the analysis, ambulatory BMD samples were kept consistent and compared to both healthy control and non-ambulatory BMD samples in our experiments. In this analysis, 1349 proteins were identified and quantified across ambulatory BMD and healthy control samples. Of these, 35 were found significantly elevated (≥1.5-fold, *p*-value ≤ 0.05) and 5 significantly decreased (≤−1.5-fold, *p*-value ≤ 0.05) in ambulatory BMD relative to controls (see supplemental Table S4). This TMT analysis identified many of the same muscle injury–related proteins detected by LFQ proteomics, along with additional proteins as shown in Table 2, albeit with different fold changes and *p*-values due to the quantitative dynamic range limitation of the TMT method. While TMT enables multiplexed analysis, often resulting in fewer missing values than LFQ, it is typically constrained by a narrower dynamic range of quantification, a limitation primarily driven by ratio compression [25].

Since we observed a difference in the urinary levels of muscle injury biomarkers between ambulatory and non-ambulatory BMD in the LFQ proteomics analysis, we sought to confirm these findings using TMT quantitative proteomics. The data showed that the levels of muscle injury proteins, particularly titin, were approximately two-fold higher in ambulatory BMD compared with non-ambulatory BMD. This difference is most likely due to ambulatory BMD individuals being more physically active and therefore experiencing greater muscle fiber breakdown than non-ambulatory BMD individuals. However, the low throughput and moderate accuracy of label-free proteome profiling, combined with the limited sample sizes that can be processed and analyzed, make it difficult to draw definitive conclusions about the most effective urinary biomarker for assessing disease stage in BMD. Therefore, validation of the mass spectrometry findings is required. Since titin appeared to be sensitive for distinguishing between ambulatory and non-ambulatory BMD in the discovery mass spectrometry data, we sought to validate this biomarker using targeted mass spectrometry and a commercially available ELISA assay targeting the N-terminal fragment of the titin protein.

### 3.2. Validation of Urinary N-Terminal Titin as a Biomarker for Becker Muscular Dystrophy

#### 3.2.1. Targeted Mass Spectrometry

Specific titin tryptic peptides to quantify and assess urinary levels of titin were selected based on the frequency of peptide detection during the initial label-free proteome profiling and optimization experiments (60 LC-MS/MS DDA runs). Thirteen tryptic peptides (see Table S6) derived from the titin protein were identified in urine samples analyzed using DDA mode. These peptides mapped to positions 42–34,322 of the protein sequence, with greater peptide coverage observed toward the N-terminal region. Of these, one peptide showed consistency in detection across runs, [_932_VPAPVEIPVTPPTLVSGLK_950_] (Light *m*/*z* = 957.5692++, Heavy *m*/*z* = 961.5763++; RT:105.19) and was selected for targeted mass spectrometry analysis using a larger sample size.

**Figure 2:**
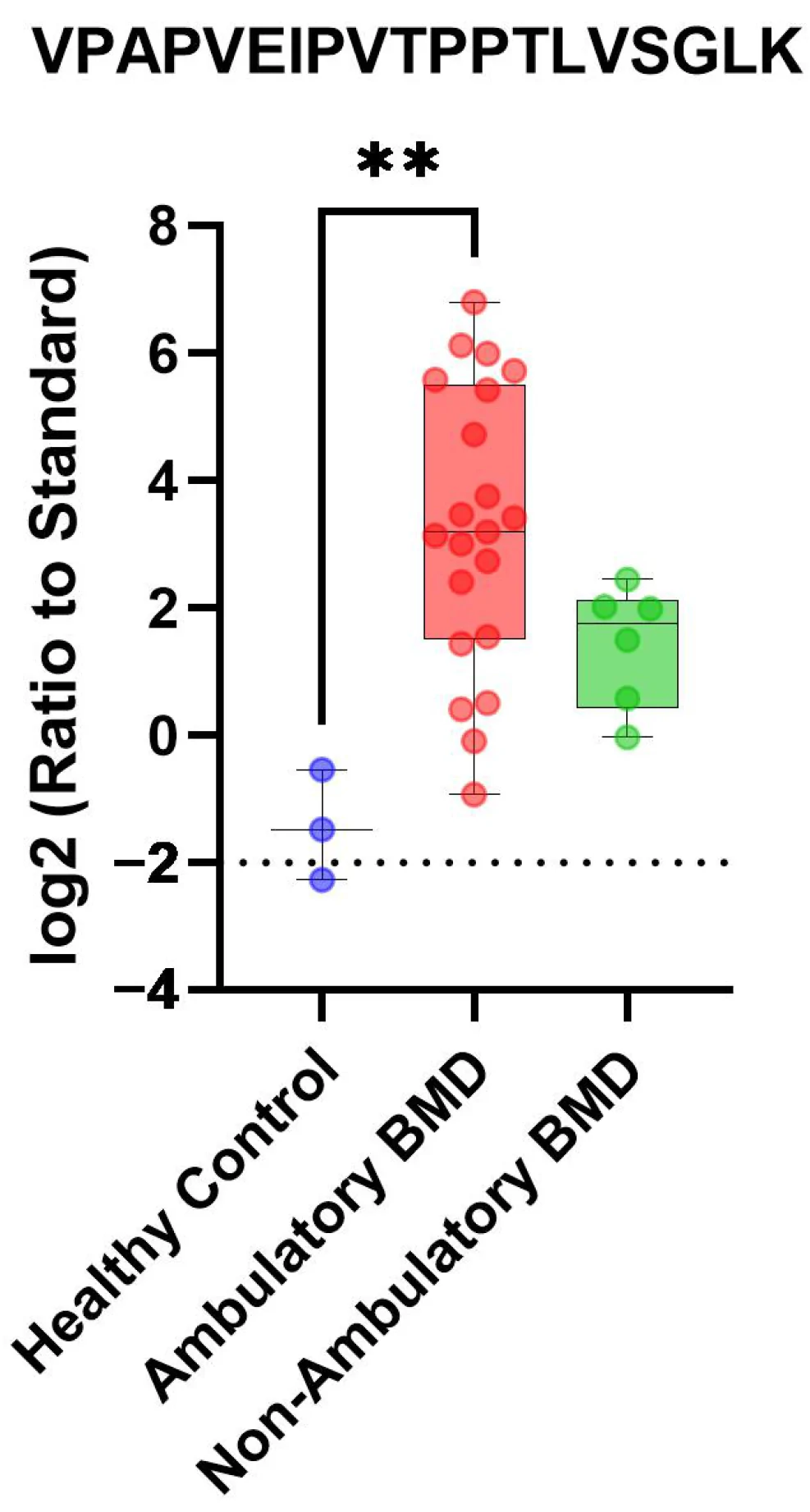
Levels of titin tryptic peptide measured by targeted mass spectrometry in urine samples of BMD patients and healthy controls. Titin tryptic peptide VPAPVEIPVTPPTLVSGLK was targeted for quantification using PRM and SILAC spike-in strategy. Data are plotted as log2 ratio of endogenous peptide to SILAC-labeled peptide. Healthy controls (*n* = 3); ambulatory BMD (*n* = 21), non-ambulatory BMD (*n* = 6). ** indicates significant *p*-value of 0.0018; reported using Tukey’s post hoc analysis.

Urine samples from ambulatory BMD (*n* = 21), non-ambulatory BMD (*n* = 6) and age-matched healthy volunteers (*n* = 3) were analyzed using our optimized targeted mass spectrometry method. Figure 2 visualizes the log2 ratios of endogenous titin peptide to SILAC standard (log2) values for the peptide VPAPVEIPVTPPTLVSGLK, demonstrating differences in urinary titin levels among ambulatory BMD and healthy controls. Using one-way ANOVA, our data showed a significant difference in the levels of urinary titin between groups (*F* = 8.397, *R*^2^ = 0.3835, *p*-value = 0.0015). Tukey’s HSD post hoc test showed significantly higher urinary titin levels in aBMD versus Cnt: (mean difference = −4.680, (95% CI −7.691 to −1.669), adjusted *p*-value = 0.0018). A secondary post hoc analysis using healthy controls as the reference group, conducted with Dunnett’s T3, indicated significantly higher levels for aBMD versus Cnt and naBMD versus Cnt (see Supplemental Figure S4). Fold change and significance were further evaluated using *t*-test analysis. A significant difference was observed between non-ambulatory BMD and healthy controls FC: 7.46, *p*-value = 0.032, whereas the comparison between ambulatory BMD versus non-ambulatory BMD and ambulatory BMD versus healthy control, showed a FC of 7.57 and 56.48 respectively, but was not statistically significant.

#### 3.2.2. Validation of Urinary N-Terminal Titin Using ELISA Assay

**Figure 3:**
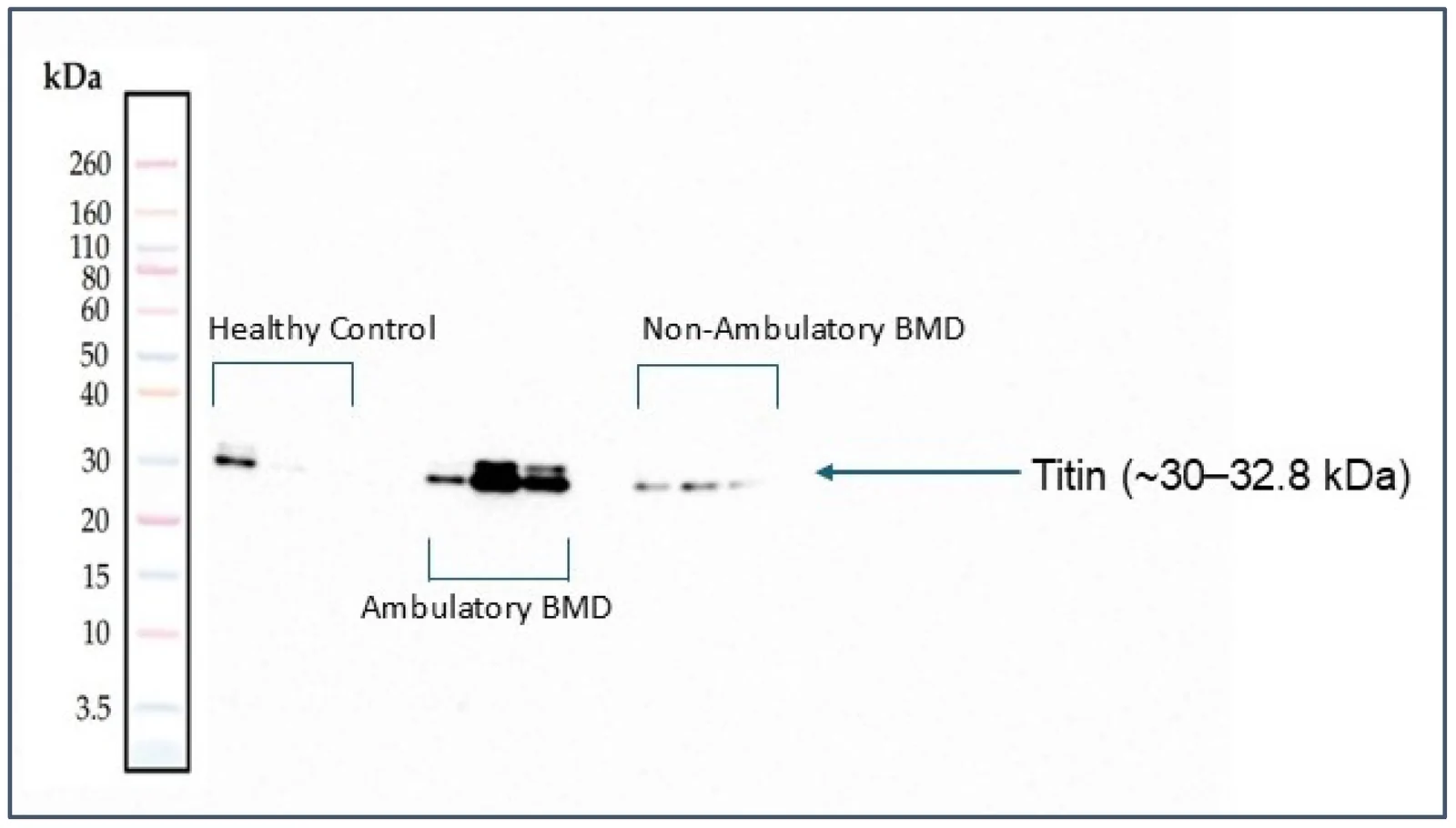
Western blot detection of N-terminal titin (~30–32.8 kDa) in urine samples of healthy controls (*n* = 3), ambulatory BMD (*n* = 3) and non-ambulatory BMD (*n* = 3). Urine aliquot containing 15 µg of total proteins was processed for Western blot analysis as described in the Methods.

We first performed a Western blot to confirm the presence of the N-terminal titin in urine samples, using antibodies recognizing the first 110 amino acids of titin protein. Samples from healthy controls (*n* = 3), ambulatory BMD (*n* = 3), and non-ambulatory (*n* = 3) were compared. As expected, the N-terminal titin fragment was detected around 30 kDa in urine samples of all six BMD patients but also in 1 of the 3 healthy controls (Figure 3). The bands were on average 8 times more intense in urine samples of ambulatory BMD compared to non-ambulatory BMD when normalized to ponceau staining, consistent with our mass spectrometry data above. One of the healthy volunteers showed the presence of the N-terminal titin fragment in urine, which could be due to intense exercise or an undiagnosed muscle condition. Unfortunately, we are unable to confirm this, as all samples were received in a de-identified manner for this study. Only age and gender information were provided, in accordance with the approved IRB protocol. Therefore, any interpretation regarding this finding remains speculative at this time. However, the analysis of urine samples from three additional healthy volunteers in this study did not reveal the presence of the N-terminal titin fragment.

**Figure 4:**
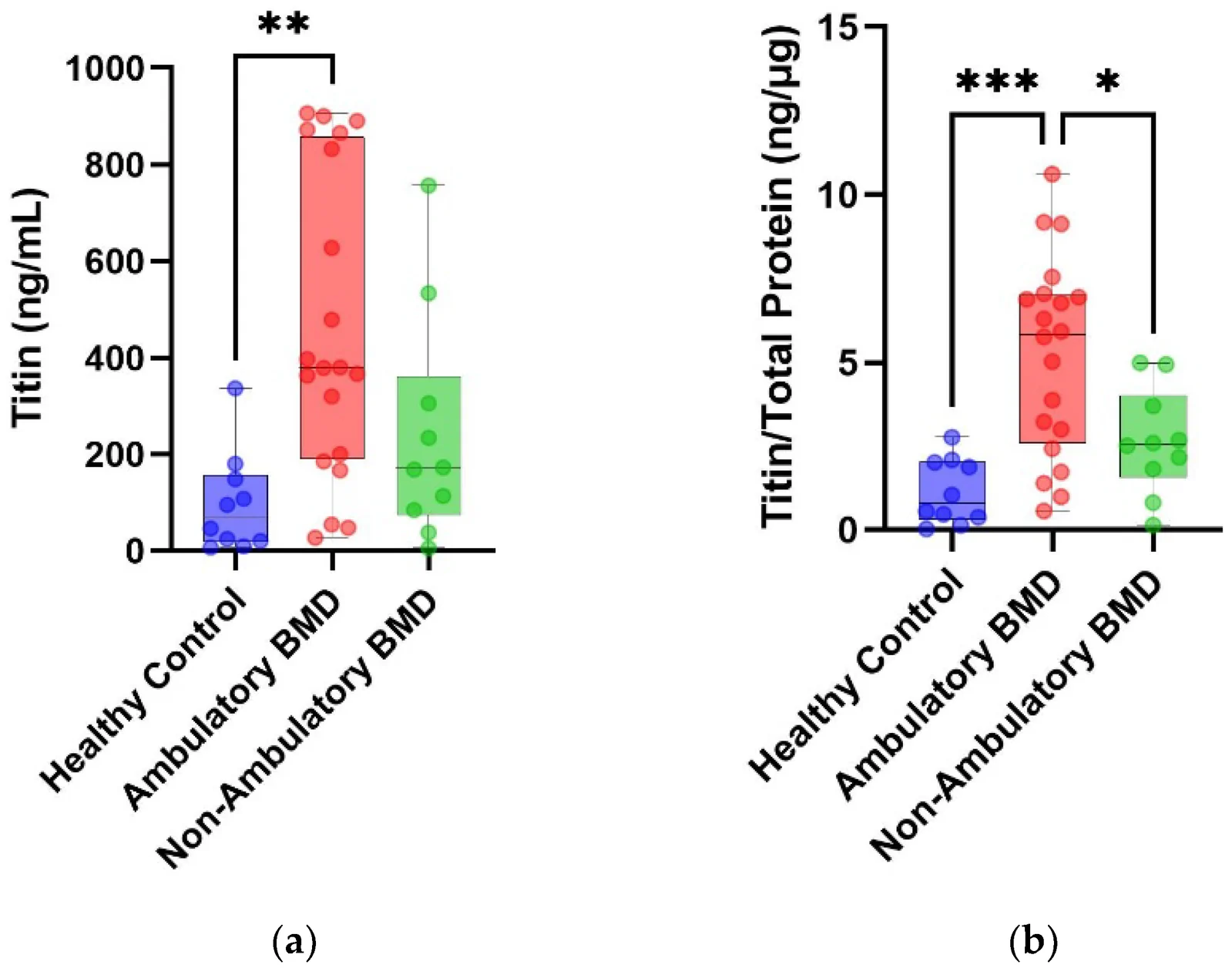
Box plots representing the levels of the N-terminal titin fragment in urine samples of ambulatory BMD (*n* = 20), non-ambulatory BMD (*n* = 10) and healthy controls (*n* = 10). (**a**) Non-normalized data; (**b**) data normalized to total protein concentration in urine samples. * *p*-value = 0.02; ** *p*-value = 0.0023; *** *p*-value = 0.0001; reported using Tukey’s HSD post hoc analysis.

Then we used a commercially available ELISA assay targeting the N-terminal fragment of titin from Immuno-Biological Laboratories, Inc. (IBL, Minneapolis, MN, USA) to validate our mass spectrometry data using a larger sample size; i.e., ambulatory BMD (*n* = 20), non-ambulatory BMD (*n* = 10) and age-matched healthy controls (*n* = 10). The results are reported with and without normalization to total protein concentration in urine and expressed in ng/mL and ng/µg total protein, respectively (Figure 4). Data were normalized to total protein concentration in urine, as creatinine levels are known to fluctuate in individuals with disease severity in DMD and BMD [24]. Since the exact size and amino acid sequence of the N-terminal titin fragments detected in BMD samples have not been fully characterized, we used an approximate number of amino acids (e.g., the first 200 amino acids), which corresponds to a calculated molecular weight of 21,358.826 g/mol for the N-terminal titin fragment used to establish the commercially available ELISA assay [23].

Figure 4a shows data without normalization to total proteins. Simple and multiple linear regression were used to examine the relationship between known standards and unknown sample values. A one-way ANOVA was performed, revealing a significant difference in the levels of urinary titin between groups (*F* = 7.187, *R*^2^ = 0.2798, *p*-value = 0.0023). Tukey’s HSD post hoc test showed significantly higher urinary titin levels in aBMD versus Cnt: (mean difference = −365.5 (95% CI −610.5 to −120.4), adj. *p*-value = 0.0023). A secondary post hoc analysis using healthy controls as the reference group was conducted with Dunnett’s T3 and indicated significantly higher levels in aBMD versus Cnt (see Supplemental Figure S5a). A significant FC of 4.74 and *p*-value = 0.0014 between aBMD and naBMD groups was also shown using *t*-test analysis.

When levels of N-terminal titin were normalized to total protein concentration and analyzed using one-way ANOVA, we observed significant differences among the group means of (*F* = 11.62, *R*^2^ = 0.3857, *p*-value = 0.0001). In Figure 4b, we found a significant difference between ambulatory BMD and healthy control and ambulatory BMD and non-ambulatory BMD, this was observed in our analysis using Tukey’s HSD post hoc analysis: aBMD versus Cnt: (mean difference = −4.079 (95% CI −6.25 to −1.91), adjusted *p*-value = 0.0001); aBMD versus naBMD: (mean difference = 2.581, (95% CI 0.414 to 4.75), adjusted *p*-value = 0.0165). A secondary post hoc analysis using healthy controls as the reference group, which was conducted with Dunnett’s T3, indicated significantly higher levels in aBMD versus Cnt and naBMD versus Cnt (see Supplemental Figure S5b). Significant fold changes were identified using *t*-test: ambulatory BMD versus healthy controls, FC: 4.56, *p*-value = 0.00022; ambulatory BMD versus non-ambulatory BMD, FC: 1.98, *p*-value = 0.015; non-ambulatory BMD versus healthy controls FC: 2.32, *p*-value = 0.020.

### 3.3. Correlation of the Levels of Urinary N-Terminal Titin with Age and Patient-Reported Outcomes

**Figure 5:**
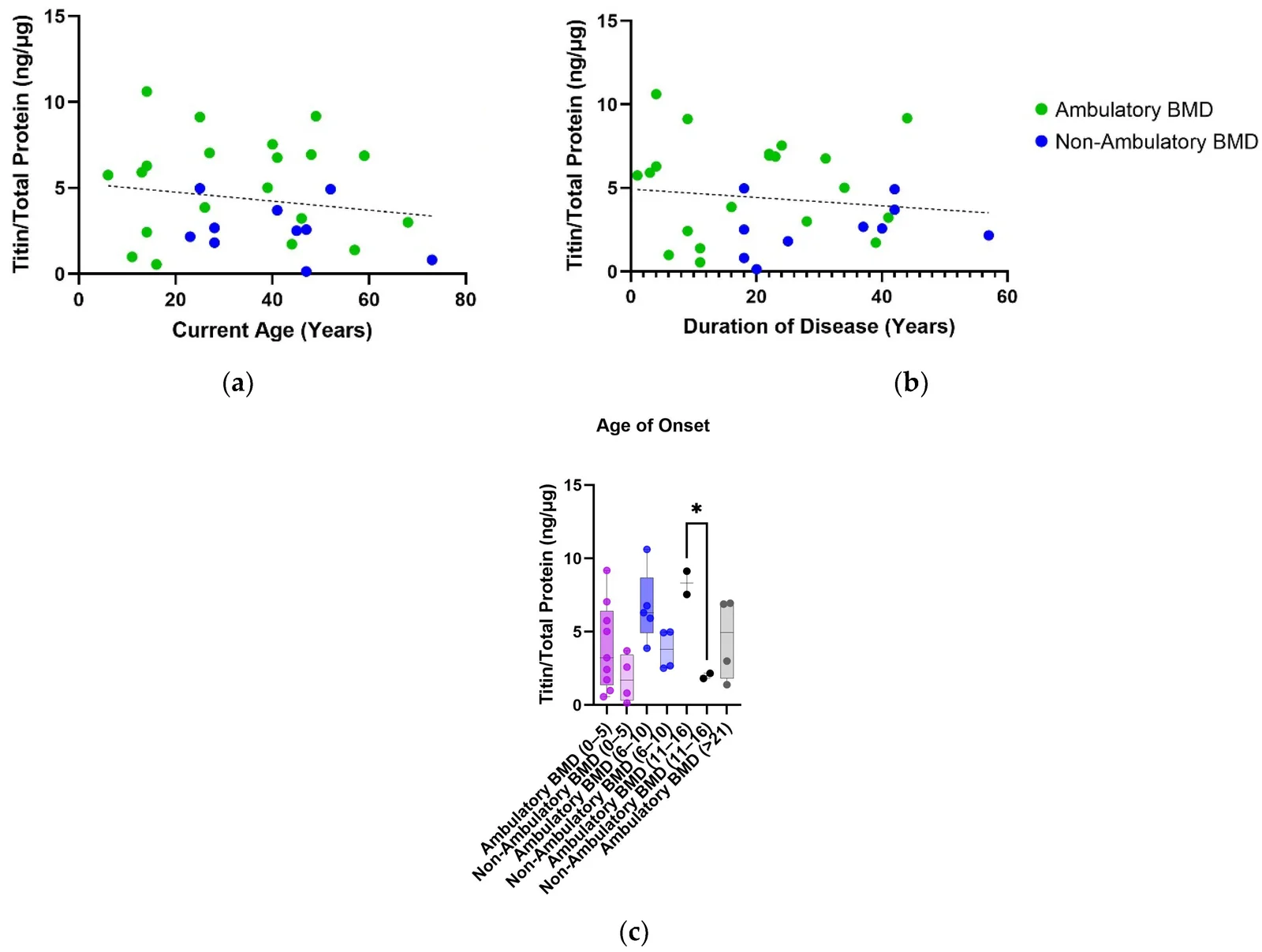
(**a**) Titin/total protein adjusted for current age, ambulatory BMD (*n* = 21), non-ambulatory BMD (*n* = 6); (**b**) Titin/total protein adjusted for duration of disease, ambulatory BMD (*n* = 21), non-ambulatory BMD (*n* = 6); (**c**) Titin/total protein comparison between the disease severities based on age of onset, ambulatory BMD (0–5) (*n* = 9), non-ambulatory BMD (0–5) (*n* = 4); ambulatory BMD (6–10) (*n* = 5), non-ambulatory BMD (6–10) (*n* = 4); ambulatory BMD (11–16) (*n* = 2), non-ambulatory BMD (11–16) (*n* = 2); ambulatory BMD (>21) (*n* = 4). * indicates significant adjusted *p*-value of 0.0425; reported using Holm–Sidak post hoc analysis.

Figure 5 shows correlation plots between levels of urinary titin and patient-reported outcomes, including age, disease duration, age of onset, and age at loss of ambulation. No significant association was observed between these outcomes and urinary N-terminal titin levels. However, when assessing the age of onset (Figure 5c), a significant difference between the disease severity groups was identified. Specifically, a significant difference was observed between the ambulatory BMD and non-ambulatory BMD in the 11–16 years age of onset group using a one-way ANOVA, (*F* = 2.87, *R*^2^ = 0.4283, *p*-value = 0.0308). Holm–Sidak’s post hoc comparisons indicated that aBMD (11–16) exceeded naBMD (11–16) by 6.34 units, with an adjusted *p*-value = 0.0425. Significant fold changes in titin levels were also observed in this comparison; a *t*-test identified a significant FC and *p*-value, 4.18 and 0.0016, respectively. Additionally, when assessing a small sample size of ambulatory BMD patients grouped based on mutation type, exon 45–47 (*n* = 5) and 45–48 (*n* = 4), a significant FC and *p*-value were obtained, FC: 2.16, *p*-value = 0.00145.

## 4. Discussion

In this study, we used global proteome profiling of urine samples collected from individuals with BMD and age-matched healthy controls to identify potential non-invasive biomarkers to assess disease stage in BMD. The BMD cohort included both ambulatory and non-ambulatory subjects. Both LFQ proteomics and TMT proteomics identified a signature of muscle injury biomarkers in urine samples of BMD subjects compared to healthy controls. This signature, consisting of TITIN, MYBPH, MYH2, MYH7, TELT, GAPDH, FLNC, and CDH13, was more pronounced in ambulatory BMD patients compared to non-ambulatory patients, most likely due to the fact that ambulatory BMD individuals have more muscle mass, are more physically active and therefore experienced greater muscle fiber breakdown than non-ambulatory BMD individuals who might have lost a lot of muscle mass and are less physically active.

Titin, also known as connectin, is the largest protein in humans and is primarily observed in skeletal and cardiac muscle. Its proteoforms, like N2B and N2BA act as a molecular spring that connects the myosin thick filaments to the Z-disk, thereby providing structural integrity to the sarcomeres [26]. In BMD, the sarcolemma is fragile due to the reduced levels of truncated dystrophin protein. This fragility leads to increased calcium influx, which is believed to activate calpains that cleave titin at the N-terminal and C-terminal side [27]. These fragment ends can then be filtered by the kidneys and excreted into the urine, while the large middle fragment remains in the blood circulation. However, a recent study showed that N-terminal fragments of titin were also found to be highly elevated in urine samples of limb–girdle muscular dystrophy recessive 1 (LGMDR1) with mutations in the calpain-3 gene compared to age-matched healthy volunteers and in Capn3-/- mice compared to wild-type mice [28]. This suggests that other mechanisms might lead to the release of the N-terminal fragment of titin into the circulation. Consistent with this possibility, our study demonstrated that titin fragments detected in the urine of BMD patients mapped to titin sequences spanning amino acid positions 42 to 34,322. This indicates that proteolytic enzymes other than calpains might be involved in titin degradation generating multiple pathogenesis-derived titin-proteoforms. Further top-down mass spectrometry studies, aimed at mapping the pathogenesis generated titin proteoforms to specific regions of the titin sequence, are needed to elucidate that these are specific to BMD, by comparing them to other muscle diseases such as DMD and limb–girdle muscular dystrophy.

BMD patients in this study were categorized based on ambulatory status. Patients who were able to walk at the time of urine collection were classified as ambulatory BMD, whereas those requiring wheelchair assistance were classified as non-ambulatory BMD. The hypothesis of this study was that ambulatory BMD patients exhibit greater active muscle contraction compared with non-ambulatory BMD patients. As a result, they are expected to retain more muscle tissue that undergoes mechanical stress during movement, leading to muscle protein breakdown and the release of muscle-derived proteins into the circulation and ultimately into the urine. As the disease progresses and patients transition to the non-ambulatory phase, irreversible muscle loss occurs and muscle tissue is gradually replaced with fatty tissue. At this stage, mobility is significantly reduced due to minimal or absent muscle contraction and decreased muscle mass. Consequently, less titin is available to be degraded in muscle tissue and subsequently detected in the circulation or in the urine. Therefore, assessing a non-invasive biomarker that directly reflects muscle wasting could provide valuable insight into a patient’s disease state, help evaluate disease severity at a given time point, and potentially allow for monitoring of disease progression as patients undergo treatment. Unfortunately, we did not have any muscle mass tests or MRIs performed on these BMD patients enrolled in this biomarker study. However, it is well known that non-ambulatory BMD patients have less muscle mass than ambulatory patients [29].

Longitudinal follow-up studies of these same patients would provide stronger insight into the hypothesis that decreases in urinary titin levels are patient-dependent and correlate with disease severity. Ideally, each patient’s N-terminal titin levels would be measured at diagnosis to establish an individual’s baseline levels and monitored over time. This could also be explored in a mouse model longitudinal study. Previous BMD mouse models have been developed specifically for the in-frame deletions of 45–47 exons (*bmx*), 52–55 (Dmd del52–55) exons, 45–48, and 45–49 [30,31,32] and these could serve as a good model to monitor disease progression and response to investigational drugs. Additional variables can be studied without concern for unethical practices using BMD mouse models by limiting medication intervention and can be customized to the mutation type for exploring trends between the disease severities. Because of this study’s limitations, including its small cohort size and even smaller groups of similar exon mutation types for consideration of medication intervention, future studies are necessary to assess potential trends such as levels of N-terminal titin peptides unique to a mutation type, impacts of medical intervention, age of onset, and duration of disease, which can be tracked by standard methods of monitoring using muscle MRI and functional tests, like the Six-Minute Walk Test (6MWT) and North Star Ambulatory Assessment (NSAA) [12,33].

Within the realm of assessing urine biomarkers for muscular dystrophies or other muscle-related diseases, the possible urinary parameters against which a biomarker value can be normalized (by expressing the biomarker level relative to that of the urinary parameter), are unclear and not standardized, particularly for BMD. Urinary creatinine is a parameter commonly used for normalization of levels of substances seen in urine samples, by expressing the level as relative to that of creatinine. In previous studies, creatinine has been used for titin normalization. Specifically for BMD, one study found that there was a higher number of non-ambulatory BMD patients who had higher levels of urinary titin/creatinine ratio overall when comparing groups of low versus high titin levels using the study’s median titin/creatinine ratio level as the threshold [24]. The results of this study are contradictory to our findings regarding assessing disease severity. This is due to the innate nature of creatinine as an indicator of overall muscle mass and an indirect indicator of muscle loss. Thus, normalization of the level of a muscle wasting biomarker according to the level of creatinine could result in skewing of the patient biomarker values.

Specific gravity has also been proposed as a urinary parameter, against which the level of a urinary biomarker can be normalized [34]. The mean specific gravity for DMD and healthy age-matched subjects found in a previous study (also found in this study in BMD patients) is 1.0200 for human urine [34]. Although it has been used before for assessing hydration and kidney function and is useful in BMD patients in the rare case of renal failure or medication side effects, specific gravity is not suitable on its own as a comparison ‘normalization’ parameter for assessing protein biomarker levels. Methods for normalization should account for the patient’s overall present pathogenesis, which can be unique to that individual due to many variables such as mutation type, age, age of onset, medication intervention, and ambulatory status.

Using total protein as a comparative parameter for normalization allows for an overall holistic biological system approach comparing the amount of biomarker protein found to total protein urine excretion. Urinary titin levels normalized to total protein were found to be more sensitive in distinguishing between ambulatory and non-ambulatory BMD patients, and between ambulatory BMD to healthy controls. Normalization of urinary titin against total protein may better account for overall protein excretion, whereas creatinine normalization can be influenced by factors such as muscle mass which is very variable among BMD patients. Furthermore, some BMD patients may take creatine as a supplement which might slightly influence the levels of creatinine. However, normalization to total urinary protein has its own limitations, including the potential influence of non-specific proteinuria and differences in diet between subjects. Hence, overall future work requires investigation of what are the ‘denominator’ parameters for normalizing urinary protein biomarkers in a larger cohort, ideally focusing on the incorporation of in tandem analysis of multiple methods of normalization to rule out other potential symptoms or comorbidities that these patients are susceptible to (in rare cases of kidney damage) [1]. These normalization methods’ results are provided in the supplemental section for reference (see Supplemental Figures S6 and S7 and Tables S7 and S8).

In terms of other muscle diseases, N-terminal titin has been elevated in the serum of sarcopenia patients, decreasing with rehab [35] and in the urine of limb–girdle muscular dystrophies, especially LGMDR1 [36], myotonic dystrophy type 1 (DM1) [13], Fukuyama congenital muscular dystrophy (FCMD) [14], idiopathic inflammatory myopathies [37], dilated cardiomyopathies (DCM) [38], and amyotrophic lateral sclerosis (ALS) [17]. In DMD, titin is currently being used to assess efficacy of dystrophin restoring therapies, specifically shown in *mdx* mice, urinary N-terminal titin falls dramatically after exon-23 skipping with a peptide-conjugated PMO, indicating titin as a pharmacodynamic marker of exon-skipping efficacy [34,39]. Additional studies with AAV-directed gene transfer methods in *mdx* mice, golden retriever muscular dystrophy (GRMD) model and humans demonstrated a good correlation between circulating titin and the levels of microdystrophin protein [19]. The accumulation of evidence on urinary titin fragments as a non-invasive biomarker suggests significant potential for assessing disease prognosis in BMD and other muscle diseases, and it could serve as a valuable tool to guide current and future drug development [40].

## 5. Conclusions

In conclusion, our study indicates that urinary N-terminal titin is a candidate in assessing disease severity and has potential in monitoring disease progression for BMD. Its non-invasive benefit and reflection of muscle loss provides direct insight into ongoing myofibrillar damage, reflecting pronounced differences in disease status. However, the inherent heterogeneity of BMD requires a shift from a “one-size-fits-all” progression criterion to an individualized baseline assessment when it comes to analyzing muscle-damage-specific biomarkers like urinary N-terminal titin. To fully validate urinary N-terminal titin’s role in monitoring disease progression, future research must incorporate a larger sample size, a collection of clinical patient outcome variables, and a long-term longitudinal design. To date, no longitudinal studies have been conducted in BMD to evaluate changes in urinary titin levels over time. However, recent studies in Duchenne muscular dystrophy (DMD), a more severe form of muscular dystrophy, have demonstrated a progressive decline in muscle injury biomarkers in longitudinal serum samples, including titin, as the disease advances. This decline correlates with the progressive loss of muscle mass. We expect that a similar pattern will be observed in BMD, with patients who experience more rapid muscle loss exhibiting a steeper decline in titin levels than those whose disease remains relatively stable over time. We also expect that therapies aimed at preserving muscle mass, but not completely curing the disease, will maintain stable levels of urinary titin over time.

## Figures and Tables

**Table 1:** Muscle-enriched proteins identified in urine samples of BMD subjects.

Accession	Entry Name	aBMD/ Cnt	*p*-Value	naBMD/ Cnt	*p*-Value	Tissue Specificity
Q8WZ42	TITIN	33	0.003	15	<0.001	heart muscle, skeletal muscle, tongue
P12883	MYH7	11	0.012	10	0.15	heart muscle, skeletal muscle, tongue
Q9UKX2	MYH2	14	0.001	13.75	<0.001	skeletal muscle, tongue
O15273	TELT	10	0.027	8.75	0.09	heart muscle, skeletal muscle, tongue
O00499	BIN1	9	0.032	1.88	0.36	skeletal muscle, tongue
Q14315	FLNC	2.47	0.01	0.88	0.83	heart muscle, skeletal muscle, tongue

Well-known muscle-related proteins that were found significantly elevated in urine samples of ambulatory BMD compared to healthy controls, included titin (TITIN), telethonin (TELT), myosin-2 (MYH2), and myosin-7 (MYH7).

**Table 2:** Muscle-enriched candidate protein biomarkers quantified using TMT method in urine samples of BMD patients and healthy controls.

Accession	Entry Name	aBMD/Cnt	*p*-Value	aBMD/ naBMD	*p*-Value	Tissue Specificity
Q8WZ42	TITIN	2.21	0.02	1.93	0.019	heart muscle, skeletal muscle, tongue
Q13203	MYBPH	2.62	0.04	1.84	0.146	skeletal muscle, tongue
Q9UKX2	MYH2	3	0.04	−1.16	0.83	skeletal muscle, tongue
O15273	TELT	2.34	0.06	1.17	0.55	heart muscle, skeletal muscle, tongue
P04406	GAPDH	1.96	0.06	1.58	0.09	skeletal muscle, tongue
P12883	MYH7	4.3	0.09	1.55	0.022	heart muscle, skeletal muscle, tongue
Q14315	FLNC	1.78	0.09	1.82	0.301	heart muscle, skeletal muscle, tongue
P55290	CDH13	−1.91	0.09	−1.26	0.137	heart muscle, skeletal muscle, tongue

Cnt: healthy controls; aBMD: ambulatory BMD; naBMD: non-ambulatory BMD; TITIN: titin; MYBPH: myosin-binding protein H; MYH2: myosin heavy chain-2; TELT: telethonin; GAPDH: glyceraldehyde-3-phosphate dehydrogenase; MYH7: myosin heavy chain-7; FLNC: filamin-C; CDH13: Cadherin-13.

## Data Availability

The mass spectrometry proteomics data have been deposited to the ProteomeXchange Consortium via the PRIDE52 partner repository with the dataset identifier PXD080765 and https://doi.org/10.6019/PXD080765.

## Supplementary Materials

The following supporting information can be downloaded at: https://www.mdpi.com/article/10.3390/proteomes14030043/s1. Figure S1. Volcano Plots of significantly elevated and decreased proteins. a. Ambulatory BMD versus Healthy Control volunteers; Fold Change (FC) ≥ 1.5; *p*-value ≤ 0.05. b. Non-Ambulatory BMD versus Healthy Control volunteers; Fold Change (FC) ≥ 1.5, ≤−1.5; *p*-value ≤ 0.05. c. Ambulatory BMD versus Non-Ambulatory BMD; Fold Change (FC) ≥ 1.5, ≤−1.5; *p*-value ≤ 0.05. Figure S2. Peptide Spectral Matches (PSMs) of Titin in BMD urine samples: Box plot showing levels of Urinary titin measured using label-free proteomic method. * indicates significant adjusted *p*-value of 0.0245; *** indicates significant adjusted *p*-value of 0.0005; reported using Dunnett’s T3 post-hoc analysis. Figure S3. Volcano Plots of significantly elevated and decreased proteins. a. Ambulatory BMD versus Healthy Control volunteers; Fold Change (FC) ≥ 1.5, ≤−1.5; *p*-value ≤ 0.05. b. Ambulatory BMD versus Non-Ambulatory BMD; Fold Change (FC) ≥ 1.5, ≤−1.5; *p*-value ≤ 0.05. Figure S4. Levels of titin tryptic peptide measured by targeted mass spectrometry in urine samples of BMD patients and healthy controls. Titin tryptic peptide VPAPVEIPVTPPTLVSGLK was targeted for quantification using PRM and SILAC spike-in strategy. Data is plotted as log 2 ratio of endogenous peptide to SILAC labeled peptide. Healthy Controls (*n* = 3); Ambulatory BMD (*n* = 21), Non-ambulatory BMD (*n* = 6). * indicates significant adjusted *p*-value = 0.0121, *** indicated significant adjusted *p*-value = 0.0005; reported using Dunnett’s T3 post-hoc analysis. Figure S5. Box plots representing the levels of the N-terminal titin fragment in urine samples of ambulatory BMD (*n* = 20), non-ambulatory BMD (*n* = 10) and healthy controls (*n* = 10). (a) prior to normalization data; (b) normalized data to total protein concentration in urine samples. * indicates significant adjusted *p*-value = 0.042; *** indicates significant adjusted *p*-value = 0.0001; **** indicates significant adjusted *p*-value = < 0.0001; reported using Dunnett’s T3 post-hoc analysis. Figure S6. Box plots represent the levels of Total Protein, Creatinine, and Specific Gravity in BMD urine samples used for normalization; (a) Total Protein (µg/mL); (b) Titin/Creatinine (mg/dL); (c) Specific Gravity. * indicates significant *p*-value of 0.014; using Tukey’s post-hoc analysis, used Dunnett’s T3 for accurate analysis for Creatinine. Figure S7. ELISA—Box plots representing the levels of the N-terminal titin fragment Normalization to Total Protein, Creatinine, and Specific Gravity in BMD urine samples: (a) Titin/Total Protein (ng/µg); (b) Titin/Creatinine (pmol/mg); (c) Titin/Specific Gravity (ng/mL); * indicates significant *p*-value: 0.0165, *** *p*-value: 0.0001 using Tukey’s post-hoc analysis. Table S1. LFQ—aBMD/Cnt, elevated proteins. Table S2. LFQ—naBMD/Cnt, elevated and decreased proteins. Table S3. LFQ—aBMD/naBMD, elevated and decreased proteins. Table S4. TMT—aBMD/Cnt, elevated and decreased proteins. Table S5. TMT—aBMD/naBMD, elevated and decreased proteins. Table S6. PRM Peptide Sequences inclusion list.; Peptides marked with (◊) was previously cited in a DMD urine study also investigating Urinary Titin (Rouillon et al. 2014, [10]). Selected based on frequency of detection in initial label-free and optimization experiments (60 LC-MS/MS DDA runs) with a cut off of the top 13 peptides. Table S7. Summary of Normalization Methods Analysis: Total Protein, Creatinine, and Specific Gravity; Data analysis was done with a one-way ANOVA, followed by Tukey’s post-hoc analysis. Table S8. ELISA—Summary of Titin Normalization Methods Analysis: Total Protein, Creatinine, and Specific Gravity; Titin/Total Protein: (Brown-Forsythe *p*-value = < 0.0001) use Tukey’s and Dunnett’s T3 for interpretation for comparison between disease severity to healthy controls; Titin/Creatinine: no significance was found; Titin/Specific Gravity: (Brown-Forsythe *p*-value = 0.131) used Tukey’s for interpretation, no significance found.

## Abbreviations

The following abbreviations are used in this manuscript:

BMD	Becker Muscular Dystrophy
DMD	Duchenne Muscular Dystrophy
aBMD	Ambulatory BMD
naBMD	Non-ambulatory BMD
Cnt	Healthy Control
CK	Creatine Kinase
LFQ	Label-Free Quantification
TMT	Tandem Mass Tag
SILAC	Stable Isotope Labeling Amino Acids by Cell Culture
ELISA	Enzyme-linked immunosorbent assay
DDA	Data dependent acquisition
PRM	Parallel Reaction Monitoring
LC-MS/MS	Liquid chromatography tandem mass spectrometry
VPAP	Tryptic titin peptide: VPAPVEIPVTPPTLVSGLK
TITIN	Titin
MYH2	Myosin heavy chain 2
MYH7	Myosin heavy chain 7
MYBPH	Myosin-binding protein H
TELT	Telethonin
BIN1	Myc-box dependent interacting protein 1
FLNC	Filamin C
GAPDH	Glyceraldehyde dehydrogenase
CDH13	Cadherin 13
TFA	Trifluoroacetic acid
ACN	Acetonitrile
FA	Formic acid
6-MWT	6-minute walk test
NSAA	NorthStar Ambulatory Assessment Score
MRI	Magnetic resonance imaging
SMA	Adult spinal muscular atrophy
DM1	Myotonic dystrophy
FCMD	Fukuyama congenital muscular dystrophy
ALS	Amyotrophic lateral sclerosis
LGMDR1	Limb–Girdle Muscular Dystrophy (specifically R1)
DCM	Dilated cardiomyopathies
PMO	Phosphorodiamidate Morpholino Oligomers
*bmx*	BMD mouse model
*mdx*	DMD mouse model
*GRMD*	Golden retriever muscular dystrophy model
PSMs	Peptide spectral matches
FDR	False discovery rate
