# Supplementary material for: Urinary Proteome Profiling by Several Methods Identifies Titin as the Most-Differentiating Noninvasive Urinary Biomarker of Disease Severity in Becker Muscular Dystrophy
Source: Proteomes. 2026 Aug 25;14(3):43. doi: 10.3390/proteomes14030043 (PMC13611649; doi:10.3390/proteomes14030043)
Supplement: Supplementary file 1 [file proteomes-14-00043-s001.zip › proteomes-4375838-supplementary.pdf]

Urinary Proteome Profiling by Several Methods Identifies Titin as the Most-differentiating Noninvasive Urinary Biomarker of Disease Severity in Becker Muscular Dystrophy

<u>Supplemental:</u>

Urine proteome profiling for discovery of candidate biomarkers

For the discovery phase we utilized two LC-MS/MS methods including label free proteome profiling method and Tandem Mass Tag (TMT) to analyze urine samples from BMD patients and age matched healthy control volunteers.

Label-Free for Global Urine Proteomic Screening

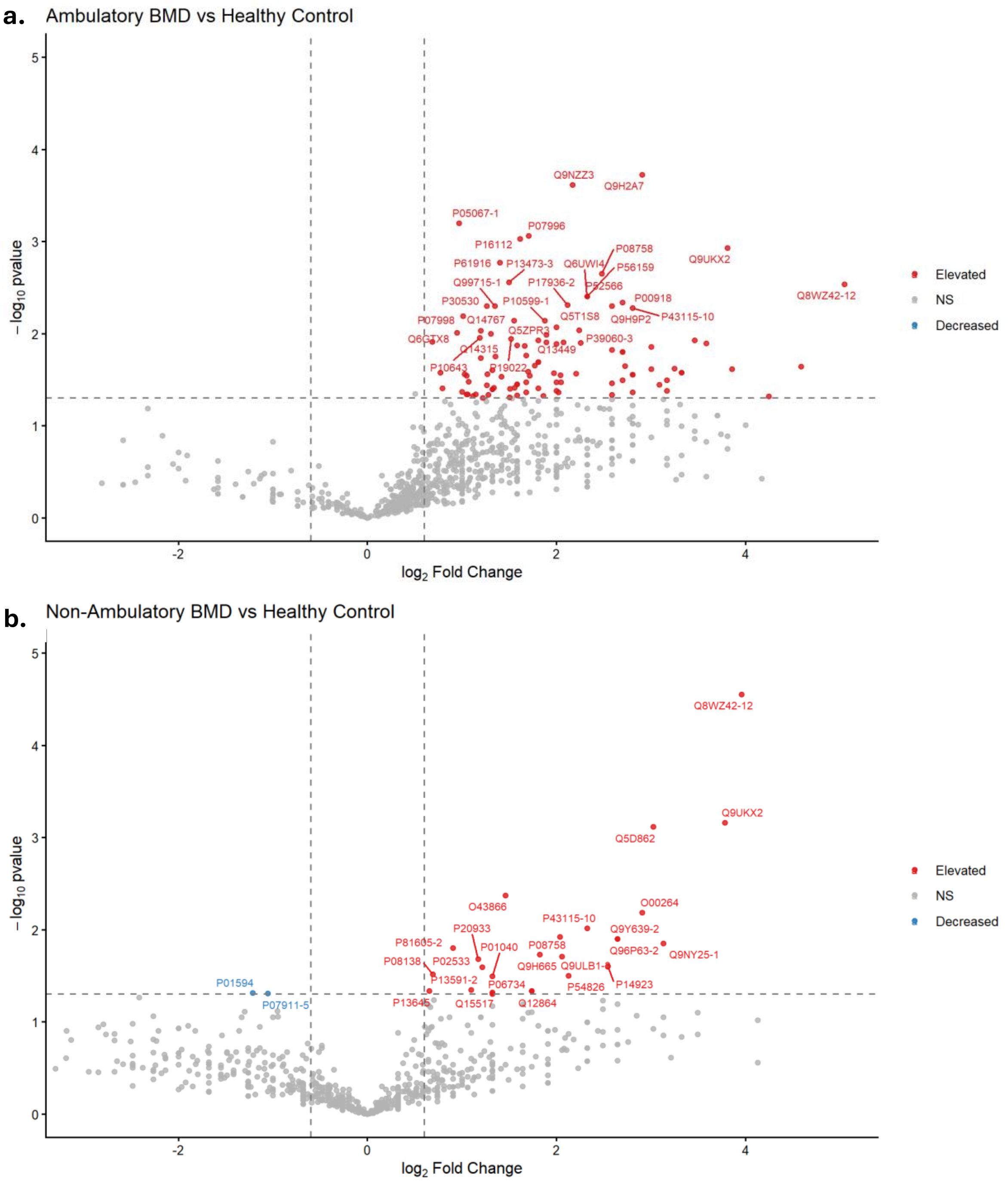

c.

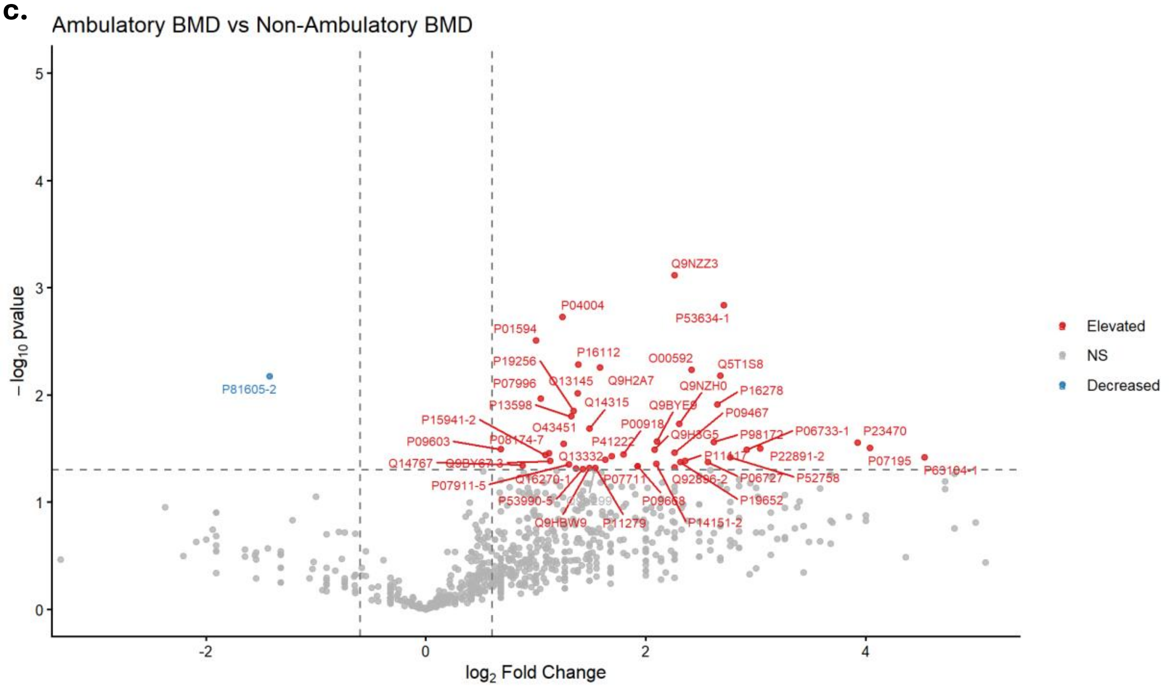

Figure S1. Volcano Plots of significantly elevated and decreased proteins.

a. Ambulatory BMD versus Healthy Control volunteers; Fold Change (FC) ≥1.5; p-value ≤ 0.05

b. Non-Ambulatory BMD versus Healthy Control volunteers; Fold Change (FC) ≥1.5, ≤ -1.5; p-value ≤ 0.05

c. Ambulatory BMD versus Non-Ambulatory BMD; Fold Change (FC) ≥1.5, ≤ -1.5; p-value ≤ 0.05

Table S1. LFQ – aBMD/Cnt, elevated proteins.

Description	Accession	aBMD/Cnt	p-value	log2fc	(-)log10pvalue
Isoform 12 of Titin	Q8WZ42-12	33.00	0.003	5.04	2.54
14-3-3 protein epsilon s	P62258-1	24.00	0.02	4.58	1.64
Ester hydrolase C11orf54	Q9H0W9	19.00	0.05	4.25	1.32
14-3-3 protein zeta/delta	P63104-1	14.50	0.02	3.86	1.61
myosin-2	Q9UKX2	14.00	0.001	3.81	2.93
Isoform 2 of Golgi apparatus protein 1	Q92896-2	12.00	0.01	3.58	1.89
Myosin-7	P12883	11.00	0.01	3.46	1.93
45 kDa calcium-binding protein	Q9BRK5-1	10.00	0.03	3.32	1.58
Telethonin	O15273	10.00	0.03	3.32	1.58
Interleukin-6 receptor subunit beta	P40189-1	9.50	0.02	3.25	1.62
Moesin	P26038	9.00	0.04	3.17	1.38
Myc box-dependent-interacting protein 1	O00499	9.00	0.03	3.17	1.50
Collectin-12	Q5KU26	8.50	0.04	3.09	1.44
Ras-related protein Rap-1b	P61224-1	8.00	0.01	3.00	1.85
14-3-3 protein beta/alpha	P31946	8.00	0.02	3.00	1.61
C-X-C motif chemokine 16	Q9H2A7	7.50	0.0002	2.91	3.73
Isoform EP3-V of Prostaglandin E2 receptor EP3 subtype	P43115-10	7.00	0.01	2.81	2.28
C-type lectin domain family 5 member A	Q9NY25-1	7.00	0.03	2.81	1.56
Receptor-type tyrosine-protein phosphatase F	P10586-1	7.00	0.03	2.81	1.56
Na(+)/H(+) exchange regulatory cofactor NHE-RF1	O14745	7.00	0.04	2.81	1.36
alpha-enolase	P06733-1	6.60	0.02	2.72	1.65
Carbonic anhydrase 2	P00918	6.50	0.005	2.70	2.34
Glycoprotein integral membrane protein 1	Q9NU53	6.50	0.02	2.70	1.80
charged multivesicular body protein 2a	O43633	6.50	0.02	2.70	1.80
Peroxiredoxin-6	P30041	6.50	0.03	2.70	1.50
Chondrolectin	Q9H9P2	6.00	0.005	2.58	2.30
Fructose-1,6-bisphosphatase 1	P09467	6.00	0.01	2.58	1.82

Nectin-1	Q15223	6.00	0.03	2.58	1.46
pituitary tumor-transforming gene 1 protein-interacting protein	P53801	6.00	0.05	2.58	1.34
annexin A5	P08758	5.57	0.002	2.48	2.65
GDNF family receptor alpha-1	P56159	5.00	0.004	2.32	2.40
Protein shisa-2 homolog	Q6UWI4	5.00	0.004	2.32	2.40
Rho GDP-dissociation inhibitor 2	P52566	5.00	0.004	2.32	2.40
Nectin-4	Q96NY8	4.78	0.01	2.26	1.90
Collagen alpha-1(XVIII) chain	P39060-3	4.71	0.01	2.24	2.04
Apolipoprotein A-IV	P06727	4.63	0.03	2.21	1.57
Charged multivesicular body protein 5	Q9NZZ3	4.50	0.0002	2.17	3.61
Isoform 2 of Insulin-like growth factor-binding protein 3	P17936-2	4.33	0.005	2.12	2.31
Neural cell adhesion molecule 1	P13591-2	4.21	0.01	2.08	1.90
Basal cell adhesion molecule	P50895	4.13	0.03	2.04	1.47
Probable serine carboxypeptidase CPVL	Q9H3G5	4.11	0.03	2.04	1.55
fibronectin	P02751	4.05	0.04	2.02	1.36
Noncompact myelin-associated protein	Q5T1S8	4.00	0.01	2.00	2.07
Isoform 3a of Neurexin-1	Q9ULB1-3	4.00	0.01	2.00	1.89
Cornifin-B	P22528	4.00	0.03	2.00	1.47
peptidyl-prolyl cis-trans isomerase A	P62937	4.00	0.04	2.00	1.38
Syntenin-1	O00560-1	3.92	0.03	1.97	1.57
Limbic system-associated membrane protein	Q13449	3.71	0.01	1.89	1.99
Isoform 7 of Osteoclast-associated immunoglobulin-like receptor	Q8IYS5-7	3.71	0.01	1.89	1.91
thioredoxin	P10599-1	3.67	0.01	1.87	2.14
Heat shock cognate 71 kDa protein	P11142-1	3.64	0.05	1.86	1.32
IGF-like family receptor 1	Q9H665	3.50	0.04	1.81	1.41
V-set and immunoglobulin domain-containing protein 4	Q9Y279	3.50	0.01	1.81	1.93
Frizzled-4	Q9ULV1	3.50	0.02	1.81	1.69
Neuroserpin	Q99574	3.50	0.02	1.81	1.69
Receptor-type tyrosine-protein phosphatase S	Q13332	3.41	0.02	1.77	1.65
Collagen alpha-3(VI) chain	P12111	3.29	0.03	1.72	1.54
thrombospondin-1	P07996	3.27	0.001	1.71	3.06
Isoform 2 of Kin of IRRE-like protein 1	Q96J84-2	3.25	0.03	1.70	1.59
CD5 antigen-like	O43866	3.20	0.02	1.68	1.76
Pancreatic secretory granule membrane major glycoprotein GP2	P55259-1	3.20	0.03	1.68	1.47
Isoform 2 of L-selectin	P14151-2	3.20	0.04	1.68	1.37
Lymphocyte function-associated antigen 3	P19256	3.17	0.01	1.66	1.87
Aggrecan core protein	P16112	3.06	0.001	1.61	3.03
N(4)-(beta-N-acetylglucosaminyl)-L-asparaginase	P20933	3.00	0.01	1.58	1.87
Growth arrest-specific protein 1	P54826	3.00	0.04	1.58	1.45
Tubulointerstitial nephritis antigen	Q9UJW2	3.00	0.04	1.58	1.45
Gamma-glutamyltranspeptidase 1	P19440	3.00	0.05	1.58	1.33
Isoform 5 of IST1 homolog	P53990-5	2.94	0.04	1.55	1.41
CD276 antigen	Q5ZPR3	2.93	0.01	1.55	2.14
CD9 antigen	P21926	2.89	0.05	1.53	1.30
Cadherin-2	P19022	2.86	0.01	1.52	1.94
Cadherin-related family member 2	Q9BYE9	2.84	0.04	1.51	1.40
Delta and Notch-like epidermal growth factor-related receptor	Q8NFT8	2.83	0.05	1.50	1.31
Isoform LAMP-2C of Lysosome-associated membrane glycoprotein 2	P13473-3	2.82	0.003	1.50	2.56
Filamin-A	P21333	2.67	0.03	1.42	1.53

Epididymal secretory protein E1	P61916	2.64	0.002	1.40	2.77
Isoform 2 of ICOS ligand	O75144-2	2.55	0.02	1.35	1.75
Collagen alpha-1(XII) chain	Q99715-1	2.55	0.01	1.35	2.30
carcinoembryonic antigen-related cell adhesion molecule 8	P31997	2.53	0.04	1.34	1.41
Podocalyxin	O00592	2.50	0.02	1.32	1.60
Adhesion G protein-coupled receptor L1	O94910	2.50	0.04	1.32	1.40
Isoform 3 of Nephronectin	Q6UXI9-3	2.50	0.04	1.32	1.40
Filamin-C	Q14315	2.47	0.01	1.30	2.00
Plexin domain-containing protein 2	Q6UX71-1	2.43	0.05	1.28	1.34
Neuronal growth regulator 1	Q7Z3B1	2.41	0.03	1.27	1.56
Ly6/PLAUR domain-containing protein 3	O95274	2.40	0.04	1.26	1.44
Tyrosine-protein kinase receptor UFO	P30530	2.40	0.01	1.26	2.30
Isoform 2 of Dynactin subunit 2	Q13561-2	2.33	0.05	1.22	1.30
Latent-transforming growth factor beta-binding protein 2	Q14767	2.30	0.01	1.20	2.03
Isoform 5 of Tenascin-X	P22105-4	2.29	0.02	1.20	1.74
Complement component C7	P10643	2.28	0.01	1.19	1.96
Isoform 2 of Mucin-1	P15941-2	2.21	0.05	1.14	1.34
Prothrombin	P00734	2.17	0.05	1.12	1.32
Isoform 3 of Cell adhesion molecule 1	Q9BY67-3	2.10	0.03	1.07	1.48
Maltase-glucoamylase, intestinal	O43451	2.08	0.05	1.06	1.34
Carboxypeptidase M	P14384	2.07	0.05	1.05	1.34
Thy-1 membrane glycoprotein	P04216	2.07	0.03	1.05	1.55
Sulfhydryl oxidase 1	O00391	2.04	0.03	1.03	1.56
Ribonuclease pancreatic	P07998	2.02	0.01	1.01	2.19
Hepatitis A virus cellular receptor 2	Q8TDQ0	2.00	0.04	1.00	1.37
Amyloid beta A4 protein	P05067-1	1.96	0.001	0.97	3.20
Leukocyte-associated immunoglobulin-like receptor 1	Q6GTX8	1.93	0.01	0.95	2.01
Fibrillin-1	P35555	1.73	0.04	0.79	1.41
Non-secretory ribonuclease	P10153	1.70	0.03	0.77	1.58
WAP four-disulfide core domain protein 2	Q14508	1.61	0.01	0.68	1.91

Table S2. LFQ – naBMD/Cnt, elevated and decreased proteins.

Description	Accession	naBMD/Cnt	p-value	log2fc	(-)log10pvalue
Isoform 12 of Titin	Q8WZ42-12	15.54	0.00003	3.96	4.55
myosin-2	Q9UKX2	13.75	0.001	3.78	3.16
C-type lectin domain family 5 member A	Q9NY25-1	8.75	0.01	3.13	1.85
Filaggrin-2	Q5D862	8.13	0.001	3.02	3.11
Membrane-associated progesterone receptor component 1	O00264	7.50	0.01	2.91	2.19
Isoform 2 of Serpin B12	Q96P63-2	6.25	0.01	2.64	1.90
Neuroplastin	Q9Y639-2	6.25	0.01	2.64	1.90
Junction plakoglobin	P14923	5.83	0.02	2.54	1.60
Isoform EP3-V of Prostaglandin E2 receptor EP3 subtype	P43115-10	5.00	0.01	2.32	2.02
Growth arrest-specific protein 1	P54826	4.38	0.03	2.13	1.50
Isoform 3a of Neurexin-1	Q9ULB1-3	4.17	0.02	2.06	1.71
annexin A5	P08758	4.11	0.01	2.04	1.92
IGF-like family receptor 1	Q9H665	3.54	0.02	1.82	1.73
Cadherin-17	Q12864	3.33	0.05	1.74	1.34
Low affinity immunoglobulin epsilon Fc receptor	P06734	3.33	0.05	1.74	1.34

CD5 antigen-like	O43866	2.75	0.004	1.46	2.37
Cystatin-A	P01040	2.50	0.03	1.32	1.49
corneodesmosin	Q15517	2.50	0.05	1.32	1.32
Agrin	O00468	2.50	0.05	1.32	1.31
Keratin, type I cytoskeletal 14	P02533	2.32	0.03	1.21	1.59
N(4)-(beta-N-acetylglucosaminyl)-L-asparaginase	P20933	2.25	0.02	1.17	1.68
Neural cell adhesion molecule 1	P13591-2	2.14	0.04	1.10	1.35
Isoform 2 of Dermcidin	P81605-2	1.88	0.02	0.91	1.80
Tumor necrosis factor receptor superfamily member 16	P08138	1.61	0.03	0.69	1.51
Keratin, type I cytoskeletal 10	P13645	1.57	0.05	0.65	1.33
Isoform 5 of Uromodulin	P07911-5	-2.07	0.05	-1.05	1.31
Immunoglobulin kappa variable 1-33	P01594	-2.32	0.05	-1.21	1.31

Table S3. LFQ – aBMD/naBMD, elevated and decreased proteins.

**Description**	**Accession**	**aBMD/naBMD**	**p-value**	**log2fc**	**(-)log10pvalue**
14-3-3 protein zeta/delta	P63104-1	23.20	0.04	4.54	1.42
L-lactate dehydrogenase B chain	P07195	16.40	0.03	4.04	1.51
Receptor-type tyrosine-protein phosphatase gamma	P23470	15.20	0.03	3.93	1.56
Isoform 2 of Vitamin K-dependent protein Z	P22891-2	8.23	0.03	3.04	1.50
alpha-enolase	P06733-1	7.54	0.03	2.92	1.49
2-iminobutanoate/2-iminopropanoate deaminase	P52758	6.80	0.04	2.77	1.42
Dipeptidyl peptidase 1	P53634-1	6.53	0.001	2.71	2.84
Noncompact myelin-associated protein	Q5T1S8	6.40	0.01	2.68	2.18
Beta-galactosidase	P16278	6.29	0.01	2.65	1.91
ephrin-B1	P98172	6.13	0.03	2.62	1.56
hemicentin-2	Q8NDA2	6.00	0.05	2.58	1.30
Apolipoprotein A-IV	P06727	5.92	0.04	2.57	1.37
Podocalyxin	O00592	5.33	0.01	2.42	2.23
Lysosomal acid phosphatase	P11117	5.12	0.04	2.36	1.39
Alpha-1-acid glycoprotein 2	P19652	4.97	0.04	2.31	1.38
G-protein coupled receptor family C group 5 member B	Q9NZH0	4.93	0.02	2.30	1.73
Charged multivesicular body protein 5	Q9NZZ3	4.80	0.001	2.26	3.11
Fructose-1,6-bisphosphatase 1	P09467	4.80	0.03	2.26	1.46
Isoform 2 of Golgi apparatus protein 1	Q92896-2	4.80	0.05	2.26	1.33
Cadherin-related family member 2	Q9BYE9	4.28	0.03	2.10	1.56
Isoform 2 of L-selectin	P14151-2	4.27	0.04	2.09	1.36
Probable serine carboxypeptidase CPVL	Q9H3G5	4.23	0.03	2.08	1.49
Cathepsin L1	P07711	3.80	0.05	1.93	1.34
Pro-cathepsin H	P09668	3.80	0.05	1.93	1.34
Carbonic anhydrase 2	P00918	3.47	0.04	1.79	1.44
Prostaglandin-H2 D-isomerase	P41222	3.22	0.04	1.69	1.43
Receptor-type tyrosine-protein phosphatase S	Q13332	3.09	0.04	1.63	1.39
C-X-C motif chemokine 16	Q9H2A7	3.00	0.01	1.58	2.25
Lysosome-associated membrane glycoprotein 1	P11279	2.91	0.05	1.54	1.32
SH3 domain-binding glutamic acid-rich-like protein 3	Q9H299	2.89	0.05	1.53	1.30

Filamin-C	Q14315	2.80	0.02	1.49	1.69
Adhesion G protein-coupled receptor L4	Q9HBW9	2.80	0.05	1.49	1.32
Isoform 5 of IST1 homolog	P53990-5	2.69	0.05	1.43	1.31
Aggrecan core protein	P16112	2.61	0.01	1.39	2.29
BMP and activin membrane-bound inhibitor homolog	Q13145	2.60	0.01	1.38	2.02
Insulin-like growth factor-binding protein 7	Q16270-1	2.57	0.05	1.36	1.31
Lymphocyte function-associated antigen 3	P19256	2.53	0.01	1.34	1.85
Intercellular adhesion molecule 2	P13598	2.50	0.02	1.32	1.80
Isoform 5 of Uromodulin	P07911-5	2.46	0.04	1.30	1.35
Maltase-glucoamylase, intestinal	O43451	2.38	0.03	1.25	1.55
Vitronectin	P04004	2.37	0.002	1.24	2.73
Pro-epidermal growth factor	P01133-1	2.28	0.05	1.19	1.29
Isoform 3 of Cell adhesion molecule 1	Q9BY67-3	2.19	0.04	1.13	1.38
Isoform 7 of Complement decay-accelerating factor	P08174-7	2.17	0.03	1.12	1.46
Isoform 2 of Mucin-1	P15941-2	2.12	0.04	1.08	1.44
thrombospondin-1	P07996	2.06	0.01	1.04	1.96
Immunoglobulin kappa variable 1-33	P01594	2.00	0.003	1.00	2.51
Latent-transforming growth factor beta-binding protein 2	Q14767	1.84	0.05	0.88	1.34
Macrophage colony-stimulating factor 1	P09603	1.60	0.03	0.68	1.49
Isoform 2 of Dermcidin	P81605-2	-2.68	0.01	-1.42	2.17

Peptide Spectrum Matches (PSMs)

A Welch's ANOVA indicated a significant difference in urinary titin levels among ambulatory BMD, non-ambulatory BMD, and healthy controls, W(2, 6.04) = 44.04, p-value = 0.0002. Shown in Figure S2., Dunnett's T3 post-hoc tests (using healthy control as reference) indicated that both ambulatory BMD group (M = 46.2) and non-ambulatory BMD group (M = 21.8) had significantly higher titin levels than healthy controls (1.4). aBMD versus Cnt: mean difference = - 44.8, 95% CI [-80.7 to -8.9], adjusted p-value = 0.0245. naBMD versus Cnt: : mean difference = - 20.35, 95% CI [-27.21 to -13.49], adjusted p-value = 0.0005.

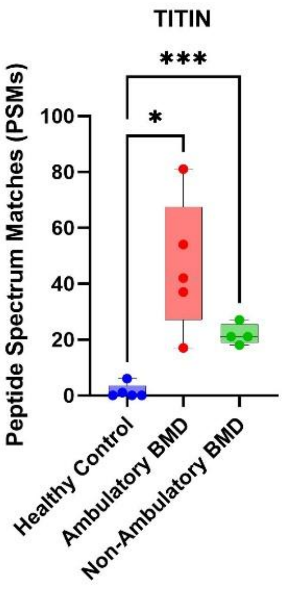

Figure S2. Peptide Spectral Matches (PSMs) of Titin in BMD urine samples: Box plot showing levels of Urinary titin measured using label-free proteomic method. * indicates significant adjusted p-value of 0.0245; *** indicates significant adjusted p-value of 0.0005; reported using Dunnett's T3 post-hoc analysis.

Tandem Mass Tag (TMT) Quantitative Proteomics

(a)

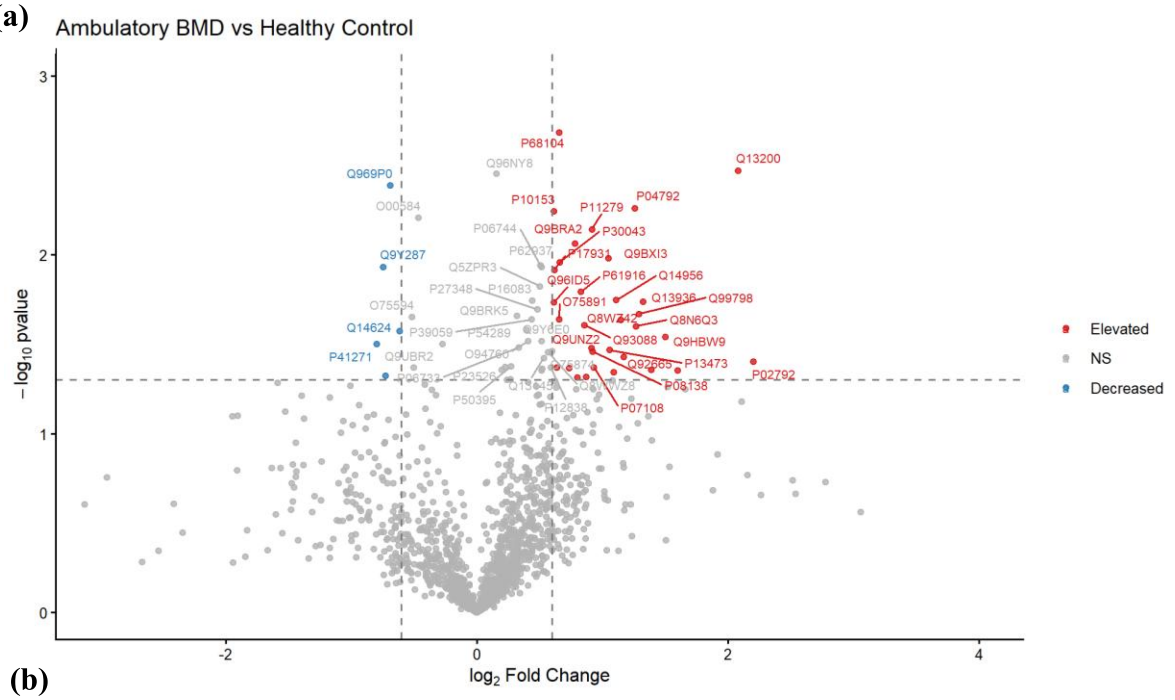

(b)

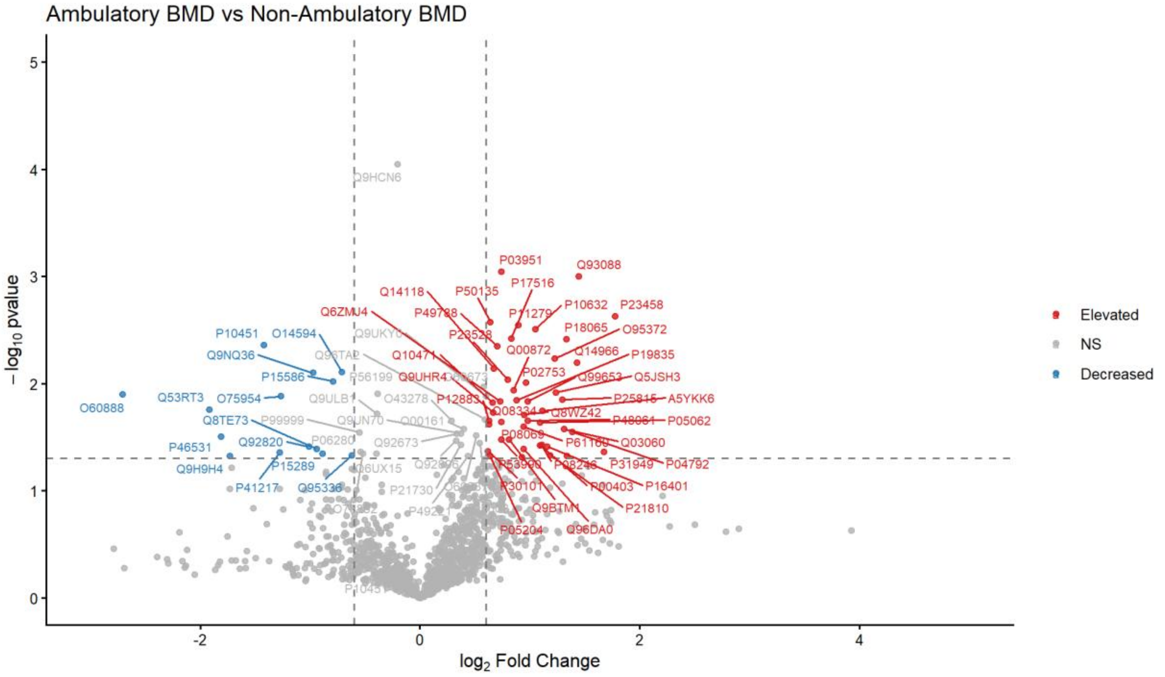

Figure S3. Volcano Plots of significantly elevated and decreased proteins.
a. Ambulatory BMD versus Healthy Control volunteers; Fold Change (FC) ≥1.5, ≤ -1.5; p-value ≤ 0.05
b. Ambulatory BMD versus Non-Ambulatory BMD; Fold Change (FC) ≥1.5, ≤ -1.5; p-value ≤ 0.05

Table S4. TMT – aBMD/Cnt, elevated and decreased proteins.

**Description**	**Accession**	**aBMD/Cnt**	**p-value**	**log2fc**	**(-)log10pvalue**
Ferritin light chain	P02792	4.60	0.04	2.20	1.40
26S proteasome non-ATPase regulatory subunit 2	Q13200	4.22	0.003	2.08	2.47

myosin-2	Q9UKX2	3.02	0.04	1.60	1.35
Adhesion G protein-coupled receptor L4	Q9HBW9	2.82	0.03	1.50	1.54
Myosin-binding protein H	Q13203	2.62	0.04	1.39	1.36
Isoform 2 of Voltage-dependent L-type calcium channel subunit alpha-1C	Q13936-2	2.50	0.02	1.32	1.74
Aconitate hydratase, mitochondrial	Q99798	2.45	0.02	1.29	1.67
CD177 antigen	Q8N6Q3	2.41	0.03	1.27	1.60
Heat shock protein beta-1	P04792	2.39	0.01	1.26	2.26
28S ribosomal protein S31, mitochondrial	Q92665	2.25	0.04	1.17	1.43
Isoform 12 of Titin	Q8WZ42-12	2.21	0.02	1.15	1.64
Transmembrane glycoprotein NMB	Q14956	2.16	0.02	1.11	1.75
UDP-glucuronosyltransferase 3A2	Q3SY77-1	2.13	0.05	1.09	1.34
Isoform 3 of Malate dehydrogenase, cytoplasmic	P40925-3	2.11	0.05	1.08	1.30
Isoform LAMP-2C of Lysosome-associated membrane glycoprotein 2	P13473-3	2.08	0.03	1.05	1.47
Cytosolic 5'-nucleotidase 1A	Q9BXI3	2.07	0.01	1.05	1.98
Isoform 5 of Acyl-CoA-binding protein	P07108-5	1.91	0.04	0.93	1.37
Tumor necrosis factor receptor superfamily member 16	P08138	1.89	0.03	0.92	1.46
Lysosome-associated membrane glycoprotein 1	P11279	1.89	0.01	0.91	2.14
Isoform 3 of NSFL1 cofactor p47	Q9UNZ2-5	1.88	0.03	0.91	1.48
Antileukoproteinase	P03973	1.83	0.05	0.87	1.32
Betaine--homocysteine S-methyltransferase 1	Q93088	1.81	0.02	0.86	1.61
Epididymal secretory protein E1	P61916	1.78	0.02	0.83	1.79
Collectrin	Q9HBJ8	1.74	0.05	0.80	1.31
Thioredoxin domain-containing protein 17	Q9BRA2	1.72	0.01	0.78	2.06
E3 ubiquitin-protein ligase RNF149	Q8NC42	1.66	0.04	0.73	1.37
flavin reductase (NADPH)	P30043	1.58	0.01	0.66	1.96
Isoform 3 of Cytosolic 10-formyltetrahydrofolate dehydrogenase	O75891-3	1.58	0.02	0.66	1.64
Elongation factor 1-alpha 1	P68104	1.58	0.002	0.66	2.68
Complement C1r subcomponent-like protein	Q9NZP8	1.56	0.04	0.64	1.37
Galectin-3	P17931	1.53	0.01	0.62	1.91
Immunoglobulin superfamily member 21	Q96ID5	1.53	0.02	0.61	1.73
Non-secretory ribonuclease	P10153	1.53	0.01	0.61	2.25
Isocitrate dehydrogenase	O75874	1.51	0.03	0.60	1.46
Neutrophil defensin 4	P12838	1.50	0.04	0.59	1.37
Inter-alpha-trypsin inhibitor heavy chain H4	Q14624-1	-1.53	0.03	-0.61	1.57
Immunoglobulin superfamily member 8	Q969P0	-1.61	0.004	-0.69	2.39
Histidine-rich glycoprotein	P04196	-1.65	0.05	-0.73	1.32
Integral membrane protein 2B	Q9Y287	-1.68	0.01	-0.75	1.93
Isoform 2 of Neuroblastoma suppressor of tumorigenicity 1	P41271-2	-1.74	0.03	-0.80	1.50

Table S5. TMT – aBMD/naBMD, elevated and decreased proteins.

**Description**	**Accession**	**aBMD/naBMD**	**p-value**	**log2fc**	**(-)log10pvalue**
Tyrosine-protein kinase JAK1	P23458	3.42	0.002	1.78	2.63

protein S100-A11	P31949	3.19	0.04	1.67	1.36
Betaine--homocysteine S-methyltransferase 1	Q93088	2.72	0.001	1.44	3.00
Zinc finger protein 638	Q14966-1	2.69	0.01	1.43	2.19
Heat shock protein beta-1	P04792	2.61	0.03	1.39	1.55
Platelet basic protein	P02775	2.53	0.05	1.34	1.32
Insulin-like growth factor-binding protein 2	P18065	2.51	0.004	1.33	2.42
Isoform 3 of cAMP-responsive element modulator	Q03060-6	2.48	0.03	1.31	1.58
Protein S100-P	P25815	2.45	0.01	1.29	1.85
WD repeat-containing protein 44	Q5JSH3	2.36	0.01	1.24	1.92
acyl-protein thioesterase 2	O95372	2.34	0.01	1.23	2.23
biglycan	P21810	2.27	0.05	1.18	1.33
Histone H1.5	P16401	2.23	0.04	1.16	1.41
CCR4-NOT transcription complex subunit 1	A5YKK6	2.16	0.02	1.11	1.74
Neutrophil elastase	P08246	2.15	0.04	1.11	1.43
Cytochrome c oxidase subunit 2	P00403	2.13	0.04	1.09	1.42
fructose-bisphosphate aldolase B	P05062	2.13	0.02	1.09	1.64
Cytochrome P450 2C8	P10632-1	2.07	0.003	1.05	2.51
Calcineurin B homologous protein 1	Q99653	1.97	0.01	0.98	1.83
Isoform Delta of Stromal cell-derived factor 1	P48061-4	1.97	0.02	0.98	1.65
Retinol-binding protein 4	P02753	1.95	0.01	0.97	2.01
Isoform 12 of Titin	Q8WZ42-12	1.93	0.02	0.95	1.71
Zymogen granule protein 16 homolog B	Q96DA0	1.92	0.04	0.94	1.39
Isoform 2 of Actin-related protein 2	P61160-2	1.92	0.03	0.94	1.60
Cystatin-C	P01034	1.90	0.05	0.93	1.31
Lysosome-associated membrane glycoprotein 1	P11279	1.86	0.003	0.90	2.54
Bile salt-activated lipase	P19835-1	1.84	0.01	0.88	1.85
Isoform 6 of Myosin-binding protein C, slow-type	Q00872-6	1.81	0.01	0.85	1.94
Aldo-keto reductase family 1 member C4	P17516	1.78	0.004	0.83	2.42
Isoform 2 of Histone H2A.J	Q9BTM1-2	1.75	0.03	0.81	1.48
Sushi, von Willebrand factor type A, EGF and pentraxin domain-containing protein 1	Q4LDE5	1.75	0.05	0.81	1.26
Cofilin-1	P23528	1.74	0.01	0.80	2.04
Coagulation factor XI	P03951	1.67	0.001	0.74	3.04
Isoform 5 of IST1 homolog	P53990-5	1.67	0.03	0.74	1.48
Interleukin-10 receptor subunit beta	Q08334	1.67	0.02	0.74	1.64
Interleukin-34	Q6ZMJ4-1	1.66	0.01	0.73	1.83
Coronin-1A	P31146	1.63	0.05	0.71	1.28
Retinoic acid receptor responder protein 1	P49788	1.63	0.004	0.70	2.35
Dystroglycan	Q14118	1.59	0.01	0.67	2.14
Brain-specific angiogenesis inhibitor 1-associated protein 2-like protein 1	Q9UHR4	1.59	0.02	0.67	1.73
Polypeptide N-acetylgalactosaminyltransferase 2	Q10471	1.58	0.02	0.66	1.82
Histamine N-methyltransferase	P50135	1.56	0.003	0.64	2.58

Non-histone chromosomal protein HMG-17	P05204	1.55	0.05	0.64	1.32
Myosin-7	P12883	1.55	0.02	0.63	1.65
Insulin-like growth factor 1 receptor	P08069	1.55	0.02	0.63	1.62
Protein disulfide-isomerase A3	P30101	1.54	0.04	0.62	1.37
Prion-like protein doppel	Q9UKY0	1.51	0.01	0.60	1.89
ATP-dependent zinc metalloprotease YME1L1	Q96TA2-1	1.51	0.02	0.59	1.67
Beta-galactoside alpha-2,6-sialyltransferase 1	P15907	1.51	0.05	0.59	1.30
6-phosphogluconolactonase	O95336	-1.54	0.05	-0.62	1.33
Neurocan core protein	O14594	-1.64	0.01	-0.71	2.11
N-acetylglucosamine-6-sulfatase	P15586	-1.73	0.01	-0.79	2.02
arylsulfatase A	P15289-1	-1.85	0.04	-0.89	1.35
Dynein heavy chain 5, axonemal	Q8TE73	-1.92	0.04	-0.94	1.39
Signal peptide, CUB and EGF-like domain-containing protein 2	Q9NQ36-1	-1.97	0.01	-0.97	2.10
Gamma-glutamyl hydrolase	Q92820	-2.02	0.04	-1.01	1.41
tetraspanin-9	O75954	-2.40	0.01	-1.27	1.88
Isoform 3 of OX-2 membrane glycoprotein	P41217-3	-2.42	0.04	-1.28	1.36
Isoform 5 of Osteopontin	P10451-5	-2.68	0.004	-1.42	2.36
Vacuolar protein sorting-associated protein 37B	Q9H9H4	-3.32	0.05	-1.73	1.32
Neurogenic locus notch homolog protein 1	P46531	-3.51	0.03	-1.81	1.51
Retroviral-like aspartic protease 1	Q53RT3	-3.79	0.02	-1.92	1.76
Isoform A of Protein CutA	O60888-2	-6.55	0.01	-2.71	1.90

Validation of Titin Biomarker Candidate

For the validation phase we utilized targeted mass spectrometry using SILAC spike and a commercially available n-terminal titin ELISA to analyze urine samples from BMD patients and age-matched healthy control volunteers.

Targeted Mass Spectrometry – SILAC

Table S6. PRM Peptide Sequences inclusion list.

Localization	Titin Tryptic Peptides	m/z	Retention Time
42-62	DGQVISTSTLPGVQISFSDGR ◊	722.0344	97.0276
99-109	AETAPPNFVQR ◊	615.31774	43.7013
932-950	VPAPVEIPVTPPTLVSGLK ◊	957.56976	101.6173
109-116	LQSMTVR	417.72844	30.4705
127-136	VTGIPTPVVK ◊	505.81567	53.612
34272-34294	VLTVACAFTGEPTPEVTWSCGGR ◊	832.39356	88.9132
82-98	ATNGSGQATSTAELLVK ◊	824.43166	51.8313
65-72	LTIPAVTK	425.77781	53.2295
34253-34271	GIPPKIEALPSDISIDEGK ◊	660.3612	50.5458
16783-16809	DGVHDIPEDAQLETAENSSVIIIPECK◊	993.81323	84.2465
185-202	ATSTAELLVQGEEEVPAK ◊	672.69955	55.8786
1004-1012	EAFAEDSGR	491.21727	25.5269
22738-22735	ATSYTITGLTENQEYK ◊	909.94187	60.4741

Peptides marked with (◊) was previously cited in a DMD urine study also investigating Urinary Titin (Rouillon et al. 2014). Selected based on frequency of detection in initial label-free and optimization experiments (60 LC-MS/MS DDA runs) with a cut off of the top 13 peptides.

A Welch's ANOVA indicated a significant difference in urinary titin levels among ambulatory BMD, non-ambulatory BMD, and healthy controls, $W(2, 7.536) = 21.12$, p-value = 0.0008. Shown in Figure S4., Dunnett's T3 post-hoc tests (using healthy control as reference) indicated that both ambulatory BMD group ($M = 3.25$) and non-ambulatory BMD group ($M = 1.41$) had significantly higher titin levels than healthy controls (-1.4). aBMD versus Cnt: mean difference = - 4.68, 95% CI [-6.63 to -2.73], adjusted p-value = 0.0005. naBMD versus Cnt: : mean difference = - 2.842, 95% CI [-4.80 to -0.89], adjusted p-value = 0.0121.

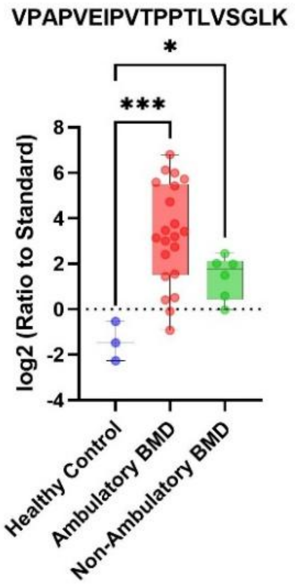

Figure S4. Levels of titin tryptic peptide measured by targeted mass spectrometry in urine samples of BMD patients and healthy controls. Titin tryptic peptide VPAPVEIPVTPPTLVSGLK was targeted for quantification using PRM and SILAC spike-in strategy. Data is plotted as log 2 ratio of endogenous peptide to SILAC labeled peptide. Healthy Controls (n = 3); Ambulatory BMD (n = 21), Non-ambulatory BMD (n = 6). * indicates significant adjusted p-value = 0.0121, *** indicated significant adjusted p-value = 0.0005; reported using Dunnett's T3 post-hoc analysis.

Validation of Urinary N-terminal Titin using ELISA assay

Analysis was first performed without normalization using Welch's ANOVA, indicated a significant difference in urinary titin levels among ambulatory BMD, non-ambulatory BMD, and healthy controls, W(2, 20.52) = 11.19, p-value = 0.0005. Shown in Figure S5a., Dunnett's T3 post-hoc tests (using healthy control as reference) indicated that ambulatory BMD group (M = 463.0) had significantly higher titin levels than healthy controls (97.53). aBMD versus Cnt: mean difference = - 365.5, 95% CI [-549.5 to -181.5], adjusted p-value = 0.0001. Following normalization, shown in Figure S5b., using Welch's ANOVA, indicated a significant difference in urinary titin levels among ambulatory BMD, non-ambulatory BMD, and healthy controls, W(2, 22.32) = 16.29, p-value = <0.0001. Dunnett's T3 post-hoc tests (using healthy control as reference) indicated that both ambulatory BMD group (M = 5.22) and non-ambulatory BMD group (M = 2.64) had significantly higher titin levels than healthy controls (1.14). aBMD versus Cnt: mean difference = - 4.079, 95% CI [-5.79 to -2.36], adjusted p-value = <0.0001. naBMD versus Cnt: : mean difference = -1.50, 95% CI [-2.94 to -0.051], adjusted p-value = 0.042.

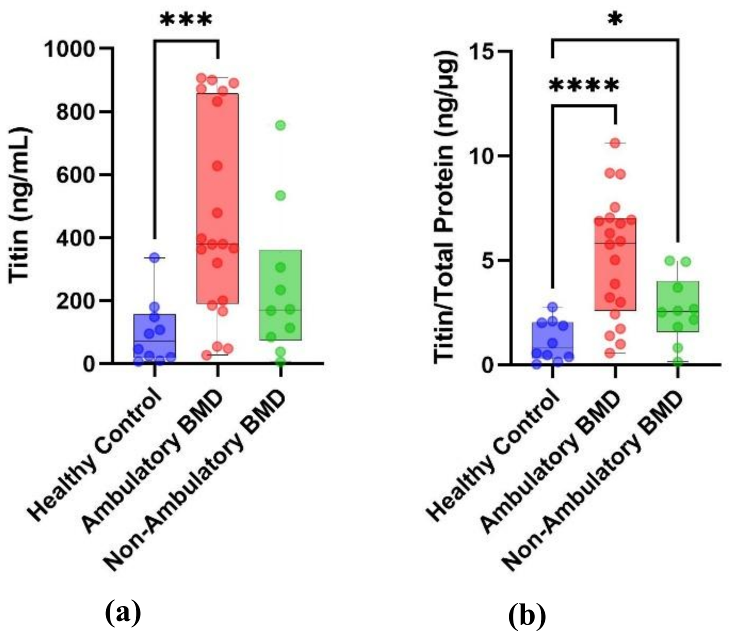

Figure S5. Box plots representing the levels of the N-terminal titin fragment in urine samples of ambulatory BMD (n = 20), non-ambulatory BMD (n = 10) and healthy controls (n = 10). **(a)** prior to normalization data; **(b)** normalized data to total protein concentration in urine samples. * indicates significant adjusted p-value = 0.042; *** indicates significant adjusted p-value = 0.0001; **** indicates significant adjusted p-value = <0.0001; reported using Dunnett's T3 post-hoc analysis.

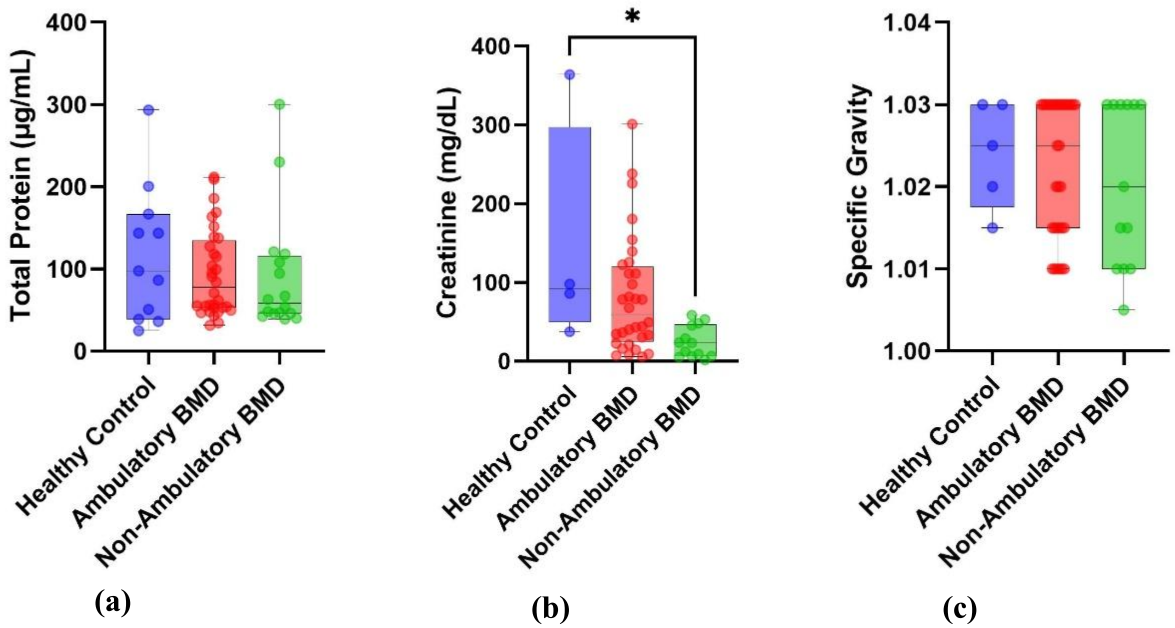

Figure S6. Box plots represent the levels of Total Protein, Creatinine, and Specific Gravity in BMD urine samples used for normalization; (a) Total Protein (µg/mL); **(b)** Titin/Creatinine (mg/dL); **(c)** Specific Gravity. * indicates significant p-value of 0.014; using Tukey's post-hoc analysis, used Dunnett's T3 for accurate analysis for Creatinine.

Table S7. Summary of Normalization Methods Analysis: Total Protein, Creatinine, and Specific Gravity

Normalization Method	Samples (n)	One-Way ANOVA p-value	aBMD/ Cnt Adjusted p-value	naBMD/ Cnt Adjusted p-value	aBMD/ naBMD Adjusted p-value
Total Protein (µg/mL)	Cnt: 11<br>aBMD: 32<br>naBMD: 16	0.566 (ns)	0.653 (ns)	0.671 (ns)	0.989 (ns)
Creatinine (mg/dL)	Cnt: 4<br>aBMD: 32<br>naBMD: 13	0.0095	0.220 (ns)	0.014	0.054 (ns)
Specific Gravity	Cnt: 5<br>aBMD: 32<br>naBMD: 13	0.695 (ns)	0.900 (ns)	0.707 (ns)	0.801 (ns)

Data analysis was done with a one-way ANOVA, followed by Tukey's post-hoc analysis.

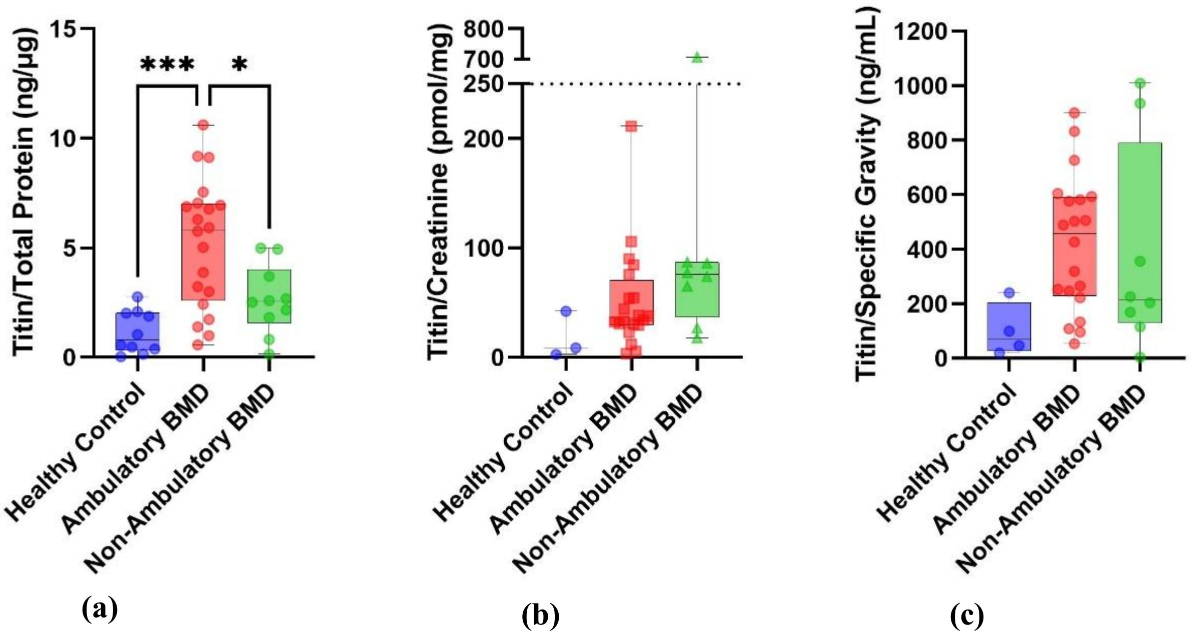

Figure S7. ELISA – Box plots representing the levels of the N-terminal titin fragment Normalization to Total Protein, Creatinine, and Specific Gravity in BMD urine samples: (a) Titin/Total Protein (ng/µg); **(b)** Titin/Creatinine (pmol/mg); **(c)** Titin/Specific Gravity (ng/mL); * indicates significant p-value: 0.0165, *** p-value: 0.0001 using Tukey's post-hoc analysis.

Table S8. ELISA – Summary of Titin Normalization Methods Analysis: Total Protein, Creatinine, and Specific Gravity

Titin/ Normalization Method	Samples (n)	One-Way ANOVA p-value	aBMD/ Cnt Adjusted p-value		naBMD/ Cnt Adjusted p-value		aBMD/ naBMD Adjusted p-value
			Tukey's	Dunnett's T3	Tukey's	Dunnett's T3	Tukey's
Titin/Total Protein (ng/µg)	Cnt: 10<br>aBMD: 20<br>naBMD: 10	0.0001	0.0001	<0.0001	0.321 (ns)	0.042	0.017
Titin/Creatinine (pmol/mg)	Cnt: 3<br>aBMD: 20<br>naBMD: 8	0.122 (ns)	0.895 (ns)	0.148 (ns)	0.296 (ns)	0.301 (ns)	0.189 (ns)
Titin/Specific Gravity (ng/mL)	Cnt: 4<br>aBMD: 20<br>naBMD: 8	0.125 (ns)	0.104 (ns)	0.002	0.250 (ns)	0.159 (ns)	0.920 (ns)

Titin/Total Protein: (Brown-Forsythe p-value = <0.0001) use Tukey's and Dunnett's T3 for interpretation for comparison between disease severity to healthy controls; Titin/Creatinine: no significance was found; Titin/Specific Gravity: (Brown-Forsythe p-value = 0.131) used Tukey's for interpretation, no significance found.
